# miR‐486‐5p Inhibits eNOS and Angiogenesis in Cultured Endothelial Cells by Targeting MAML3

**DOI:** 10.1111/jcmm.70589

**Published:** 2025-05-27

**Authors:** Adrianna Douvris, Ali Maadelat, Christopher J. Porter, Dylan Burger, Kevin D. Burns

**Affiliations:** ^1^ Division of Nephrology, Department of Medicine and Kidney Research Centre, Ottawa Hospital Research Institute University of Ottawa Ottawa Ontario Canada; ^2^ Department of Cellular and Molecular Medicine University of Ottawa Ottawa Ontario Canada; ^3^ Ottawa Bioinformatics Core Facility Ottawa Hospital Research Institute Ottawa Ontario Canada

**Keywords:** angiogenesis, endothelial cells, miRNA, miRNA pulldown, RNA‐sequencing

## Abstract

Kidney ischemia–reperfusion (I/R) is associated with endothelial injury. Administration of miRNA (miR)‐486‐5p protects against rat kidney I/R injury, with localisation to capillary endothelial cells, although it inhibits I/R‐induced endothelial nitric oxide synthase (eNOS) protein expression. Here, we studied the effect of miR‐486‐5p on eNOS and endothelial cell function and determined its mRNA targets. Human umbilical vein endothelial cells (HUVECs) were transfected with the miR‐486‐5p mimic and assayed for proliferation, migration and network formation. Biotinylated miR‐486‐5p was transfected for pulldown of bound mRNA, followed by RNA sequencing. miR‐486‐5p markedly decreased eNOS mRNA and protein in HUVECs (*p* < 0.001) and decreased eNOS protein in human pulmonary microvascular endothelial cells (*p* < 0.05), although eNOS was not a direct target of miR‐486‐5p. miR‐486‐5p inhibited angiogenesis, which was rescued with eNOS plasmid transfection. RNA sequencing of biotinylated miR‐486‐5p pulldown RNA revealed highly significant enrichment in predicted targets FOXO1, FOXP1, TNFSF4, MAML3 and CELSR3, and in the non‐predicted target SPCS2. RT‐qPCR validated these transcripts as inhibited by miR‐486‐5p. While silencing of FOXO1 had no impact on eNOS protein, MAML3 silencing inhibited eNOS levels. miR‐486‐5p inhibits angiogenesis in endothelial cells via eNOS down‐regulation, which involves selective targeting of MAML3. These data support a novel pathway regulating endothelial cell function.

## 1 Introduction

MicroRNAs (miRNAs) are small non‐coding RNAs, averaging 22 nucleotides in length, and are evolutionarily conserved post‐transcriptional regulators of genes involved in key biological processes including cell differentiation, growth and proliferation [1]. More than 60% of human protein‐coding genes are estimated to be miRNA targets [2]. miRNAs primarily regulate protein expression by binding the 3′ untranslated region (UTR) of target mRNA to induce post‐transcriptional gene silencing via mRNA degradation or inhibition of translation [1]. However, other binding sites have been identified within 5′ UTRs, introns and exons of mRNAs as well as non‐mRNA targets including long non‐coding and circular RNAs [3]. In addition, individual miRNA exhibit distinct target binding patterns [3]. Not surprisingly, studies aimed at identifying miRNA–mRNA interactions have identified hundreds to thousands of targets per miRNA [4, 5, 6].

miR‐486‐5p is a muscle‐enriched miRNA that regulates signalling pathways involved in apoptosis, cell proliferation, migration and angiogenesis [7]. Pre‐clinical studies have demonstrated that miR‐486‐5p improves muscle function in muscular dystrophy [8], protects against skeletal muscle wasting [9, 10] and cardiac ischemia–reperfusion (I/R) injury [11], and attenuates pulmonary [12] and cardiac fibrosis [13]. We previously showed that both direct miR‐486‐5p administration and exosomal transfer of miR‐486‐5p protected against kidney I/R injury in mice associated with down‐regulation of its target gene phosphatase and tensin homologue (PTEN) and genes involved in apoptosis and inflammation [14, 15]. miR‐486‐5p administered early to rats with kidney I/R localised to peritubular capillary endothelial cells and prevented development of late kidney fibrosis and systemic endothelial dysfunction after ischemic kidney injury [16].

Despite its protective short‐ and long‐term effects in kidney I/R injury, in rats miR‐486‐5p unexpectedly inhibited I/R‐induced upregulation of kidney endothelial nitric oxide synthase (eNOS) [16], suggesting potential adverse effects on vascular function. In this regard, whether miR‐486‐5p directly affects angiogenesis is unclear. Administration of miR‐486‐5p to cultured human umbilical vein endothelial cells (HUVECs) inhibited its target CADM1, resulting in increased cell permeability and invasion, without affecting angiogenesis [17], while in HUVEC spheroids, miR‐486‐5p inhibited sprouting angiogenesis [18]. In contrast, exosomal miR‐486‐5p promoted angiogenesis in animal models of myocardial infarction [19], cutaneous wound healing [20] and ischemic stoke [21]. Consequently, the effects of miR‐486‐5p on endothelial cell function are unclear and its mRNA targets remain undefined. Here, we studied the effect of miR‐486‐5p on eNOS and endothelial cell function in vitro. Experiments were conducted in normoxic endothelial cells and with exposure to hypoxia‐re‐oxygenation (H/R) to mimic I/R injury from our in vivo studies [14, 16]. We also conducted a biotinylated miRNA pulldown assay to identify mRNA targets for miR‐486‐5p in cultured endothelial cells.

## 2 Methods

### 2.1 Cell Culture

Human umbilical vein endothelial cells (HUVECs) were purchased from American Type Culture Collection (PCS‐100‐013, ATCC, via Cedarlane Corp., Burlington, ON, Canada), and cultured at 37°C in 5% CO_2_ in EBM2 medium supplemented with microvascular growth factors and 2% FBS (Lonza, Basel, Switzerland, catalogue # CC‐3156). HUVECs between Passages 4 and 7 were used for all experiments. Human pulmonary microvascular endothelial cells (HPMECs) were purchased from ScienCell (Catalogue # 3000, Carlsbad, CA, USA) and cultured at 37°C in 5% CO_2_ in Endothelial Cell Medium (ScienCell catalogue # 1001), supplemented with 5% FBS, growth supplement, and penicillin/streptomycin. Only HPMECs between Passages 4 and 6 were used. For all functional assays and target validation experiments, HUVECs were transfected with 1 nM miRVana miR‐486‐5p mimic or scramble (scb) miRNA in lipofectamine RNAiMax (Thermo Fisher Scientific, Waltham, MA, USA). For miR‐486‐5p inhibition, HUVECs were transfected with 10 nM miRVana miR‐486‐5p inhibitor (Thermo Fisher Scientific). For gene silencing, HUVECs were transfected with 5 nM eNOS siRNA, 5 nM FOXO1 siRNA or 10 nM MAML3 siRNA (SilencerSelect, Thermo Fisher).

For H/R experiments, HUVECs were transfected with miR‐486‐5p or scb miRNA and after 24 h were subjected to hypoxia (1% O_2_, 5% CO_2_, 94% N_2_) for 24 h, followed by re‐oxygenation in standard culture conditions for up to 24 h.

### 2.2 Immunoblot and Phospho‐Kinase Array

At 48 h post transfection, HUVECs were lysed in radioimmunoprecipitation assay (RIPA) buffer or nuclear and cytoplasmic extraction (NETN) buffer (latter specifically for MAML3 immunoblot). Lysates were resolved by SDS‐polyacrylamide gel electrophoresis, transferred to nitrocellulose membranes, blocked in 5% milk for 1 h and incubated for 16 h at 4°C with primary antibody against eNOS (#32027), phospho‐eNOS (#9571), FOXO1 (#2880), PTEN (#9552) (all 1:1000, Cell Signaling, Whitby, ON, Canada) or MAML3 (NB100‐2129; 1:1000; Novus Biologicals, Toronto, ON, Canada). Membranes were incubated with primary antibody against glyceraldehyde‐3‐phosphate dehydrogenase (GAPDH,1:5000, Cell Signaling, #2118) for 1 h at room temperature. Washed membranes were incubated with horseradish peroxidase‐conjugated anti‐rabbit secondary antibody (1:5000, Abcam, Toronto, ON, Canada) for 1 h at room temperature and visualised by chemiluminescence using the Alphaimager System (Alpha Innotech, San Leandro, CA, USA) or the ChemiDoc Imaging System (Bio‐Rad, Mississauga, ON, Canada). Densitometry was performed using the ImageJ software (NIH, Bethesda, MD, USA).

Screening for relative levels of phosphorylated protein kinases in HUVEC lysates from miR‐486‐5p or scb miRNA‐transfected cells was performed using the Proteome Profiler Phospho‐Kinase array kit as per manufacturer's recommendations (Catalogue # ARY003C, R&D Systems Inc., Burlington, ON, Canada). Images were obtained using the ChemiDoc Imaging System with Image Lab Software (Bio‐Rad).

### 2.3 Luciferase Reporter Assay

Reporter vectors containing Firefly and Renilla Luciferases with wild‐type or mutant eNOS 3′ UTR were obtained from Genecopeia (Rockville, MD, USA). The pEZX‐MT06‐eNOS 3′ UTR‐f/rLuc or mutant eNOS 3′ UTR‐f/rLuc vectors (50 ng) were transfected into HUVECs in a 96‐well plate with Lipofectamine 3000 alone, or with 10 nM miR‐486‐5p mirVana mimic (Thermo Fisher). Luciferase activity was determined after 24 h using a Dual Luminescence assay kit (Genecopeia) by an Orion II microplate luminometer (Berthold Detection Systems, Pforzheim, Germany).

### 2.4 5‐Bromo‐2′‐Deoxyuridine (BrdU) Cell Proliferation Assay

Cell proliferation was evaluated by measuring BrdU incorporation during DNA synthesis (Cell Proliferation ELISA, BrdU (colorimetric), Roche, Laval, QC, Canada) as per the manufacturer's protocol. HUVECs were seeded at a density of 7.5 × 10^3^ cells per well (normoxia) or 5 × 10^3^ cells per well (H/R) in a 96‐well plate and transfected with 1 nM miR‐486‐5p, 1 nM scb miRNA, or 5 nM eNOS siRNA in lipofectamine RNAiMax, or with lipofectamine alone. After 24 h, the media was replaced. Normoxic cells were incubated for another 24 h, and then labelled with 10 μM BrdU. For H/R, the cells were then placed in 1% O_2_ for 24 h, followed by re‐oxygenation for 24 h, and then labelled with 10 μM BrdU. Normoxic and H/R HUVECs were incubated for 2 h at 37°C for labeling. The labeling medium was removed, cells were dried at 60°C for 1 h, fixed, incubated with anti‐BrdU for 90 min, washed and incubated with substrate solution. After 15 min, absorbances were read at 370 nm with reference wavelength 492 nm. The data are presented as absorbance 370‐nm minus absorbance 492 nm, normalised relative to untreated controls.

### 2.5 Scratch Wound Assay

HUVECs were seeded at a density of 1 × 10^4^ cells per well onto a 96‐well ImageLock plate (Sartorius, Ann Arbor, MI, USA) coated with an attachment factor (Thermo Fisher Scientific). Cells were transfected with 1 nM miR‐486‐5p, 1 nM scb miRNA, or 5 nM eNOS siRNA in Lipofectamine RNAiMax or with Lipofectamine alone for 24 h. After another 24 h of normoxia or 24 h of hypoxia (1% O_2_), a WoundMaker (Sartorius) was used to create a uniform scratch in each well as per the manufacturer's protocol. The plate was placed in the Incucyte S3 Live‐Cell Analysis System (Sartorius), and images for cell migration were acquired every 2 h for 24 h. Wound area was determined using Image J [22].

### 2.6 Matrigel Network Formation Assay

HUVECs were seeded into six‐well plates and transfected with 1 nM miR‐486‐5p, 1 nM scb miRNA or 5 nM eNOS siRNA in Lipofectamine RNAiMax. To determine whether eNOS rescues in vitro network formation, HUVECs were reverse‐transfected with miR‐486‐5p and eNOS plasmid together or with miR‐486‐5p and mutated eNOS S1179A plasmid. pcDNA3‐eNOS‐GFP [23] and pcDNA‐eNOS S1179A [24] were purchased from Addgene (# 22444 and #22485). In vitro network formation assays were performed 48 h post transfection. Briefly, each well of a 96‐well plate was coated with 50 μL of Matrigel (Corning, Bedford, MA, USA) and incubated at 37°C for 30 min for polymerisation. HUVECs were collected by trypsinisation, counted using Trypan Blue with a haemocytometer, and seeded at a density of 1.5 × 10^4^ cells per well. At 4 h, 8 h and 24 h post seeding, Tag Image File Format (TIFF) images of capillary‐like networks were captured using a Zeiss Axio Image M2 microscope equipped with a digital camera. For hypoxia, cells plated onto Matrigel were incubated in 1% O_2_ for 8 h post seeding, followed by imaging. Images were not quantified at 24 h post seeding (16 h of re‐oxygenation) due to significant cell loss in Matrigel‐coated plates. Images were processed using the Angiogenesis function of ImageJ [25].

### 2.7 Biotinylated miRNA Pulldown

3′ biotinylated miR‐486‐5p and 3′ biotinylated cel‐miR‐67 negative control were purchased from Horizon Discovery Biosciences (Waterbeach, Cambridge, UK). HUVECs were seeded into 10‐cm^2^ plates. The pulldown protocol was adapted from Wani et al. [26] and Martin et al. [6]. 3′ Bi‐miR‐486‐5p or 3′‐Bi‐cel‐miR were transfected in Lipofectamine RNAiMax at a final concentration of 10 nM. After 24 h, cells were lysed in ice‐cold hypotonic lysis buffer (10 mM KCl, 1.5 mM MgCl_2_, 10 mM Tris–HCl (pH 7.5), 5 mM dithiothreitol (DTT), 0.5% Igepal CA‐630, 60 U/mL Superase‐In RNase inhibitor (Thermo Fisher Scientific) and 1× protease inhibitor (MilliporeSigma, Oakville, ON, Canada)). Lysates were placed on dry ice for 5 min and allowed to thaw. Lysates were then centrifuged at 12,000 × g for 2 min at 4°C to clear debris. The supernatants were transferred to clean 1.5‐mL microfuge tubes, and NaCl was added to a final concentration of 1 M.

Streptavidin magnetic beads (Dynabeads MyOne Streptavidin C1, Thermo Fisher Scientific) were prepared on the day of transfection. 125 μL of MyOne C1 beads (per 600 pmol biotinylated RNA) was washed with 1x bead binding and wash buffer (5 mM Tris–HCl (pH 7.5), 0.5 mM EDTA, 1 M NaCl), and made RNase‐free by incubating in Solution A (0.1 M NaOH, 0.05 M NaCl), then in Solution B (0.1 M NaCl) [26]. Beads were blocked overnight at 4°C with 1 mg/mL ultrapure bovine serum albumin and 1 mg/mL yeast tRNA (Thermo Fisher Scientific). The pre‐blocked beads were incubated with lysate supernatant for 30 min at room temperature and then washed in hypotonic lysis buffer plus 1 M NaCl. After the last wash, the beads were resuspended in 100 μL nuclease‐free water for RNA isolation.

### 2.8 RNA Isolation and RNA Sequencing

RNA was isolated with the miRNeasy micro kit (Qiagen Inc., Toronto, ON, Canada) with modifications to purify RNAs > 200 nucleotides. Briefly, 700 μL of Qiazol was added to the beads, followed by 140 μL of chloroform. After incubation at room temperature for 3 min, the samples were centrifuged at 12,000 × g for 15 min at 4°C. For each sample, the aqueous phase was transferred to a new 1.5‐mL microfuge tube containing 1× volume of 70% ethanol. The mixture was then transferred to an RNeasy micro‐spin column and purified according to the manufacturer's protocol. The RNA was eluted in 25 μL of nuclease‐free water. RNA sample quality assessment was performed with the Fragment Analyser (Agilent) and concentration measured with the Qubit 3.0 (Thermo Fisher Scientific). Next‐generation sequencing libraries were prepared with the Ultra II directional RNA kit (New England Biolabs Ltd., Ipswich, MA, USA) using 15 ng of RNA input. Sequencing (RNA‐Seq) was performed with the Nextseq 2000 P2 100 cycle flow cell (Illumina, San Diego, CA, USA).

### 2.9 Bioinformatics

After sequencing of pulldown RNA, the libraries were quantified using salmon v1.10.1 [27] in version 3.14.0 of the nf‐core RNAseq pipeline [28]. The gene/sample count matrix generated from salmon results was loaded into the R statistical analysis package and filtered to remove genes with no detected reads in any sample. Differential expression between biotinylated miR‐486‐5p and biotinylated cel‐miR‐67 pulldown RNA was evaluated using DESeq2 [29]. Principal component analysis (PCA) was performed using the DESeq2 plotPCA function. Given the expected lower level of biological variability between replicates, the alpha (FDR/q‐value) cut‐off parameter was set to 0.01. Raw fold changes were moderated using the ‘apeglm’ method for lfc shrinkage [30] in DESeq2; the presented log_2_FoldChange values represent these shrunken log fold change estimates. Significantly differentially expressed genes were identified using an adjusted *p* value (padj; Benjamin–Hochberg corrected *p* value) cut‐off of 0.01. Predicted targets of miR‐486‐5p were obtained from the miRDB target prediction database [31, 32] (downloaded 2024‐06‐27).

### 2.10 Real‐Time qPCR

Total RNA was isolated from HUVECs 24 h post transfection using the miRNeasy micro kit (Qiagen). Reverse transcription and real‐time qPCR for eNOS, FOXO1, FOXP1, TNFSF4, CELSR3, MAML3, SPCS2 and PTEN were performed via TaqMan Gene Expression Assays (Life Technologies Inc., Toronto, ON, Canada) using the Applied Biosystems 7300 real‐time PCR system (Foster City, CA, USA). Endogenous GAPDH was used for normalisation of mRNA levels. The relative levels of genes of interest were calculated using the 2^−ΔΔCt^ method [33].

### 2.11 Statistical Analyses

All experiments were performed in duplicate or triplicate technical replicates, with at least three biological replicates. Results are expressed as mean ± SEM, and statistical comparisons were conducted using one‐ or two‐way analysis of variance (ANOVA) with Tukey's post‐test as appropriate. Statistical analyses were performed with GraphPad Prism 10 (GraphPad Software Inc., San Diego, CA, USA). Statistical significance was set at *p* < 0.05. Statistical analysis of RNA‐seq results is described above in ‘Bioinformatics’.

## 3 Results

### 3.1 miR‐486‐5p Reduces eNOS mRNA and Protein Levels in Cultured Endothelial Cells

**FIGURE 1:**
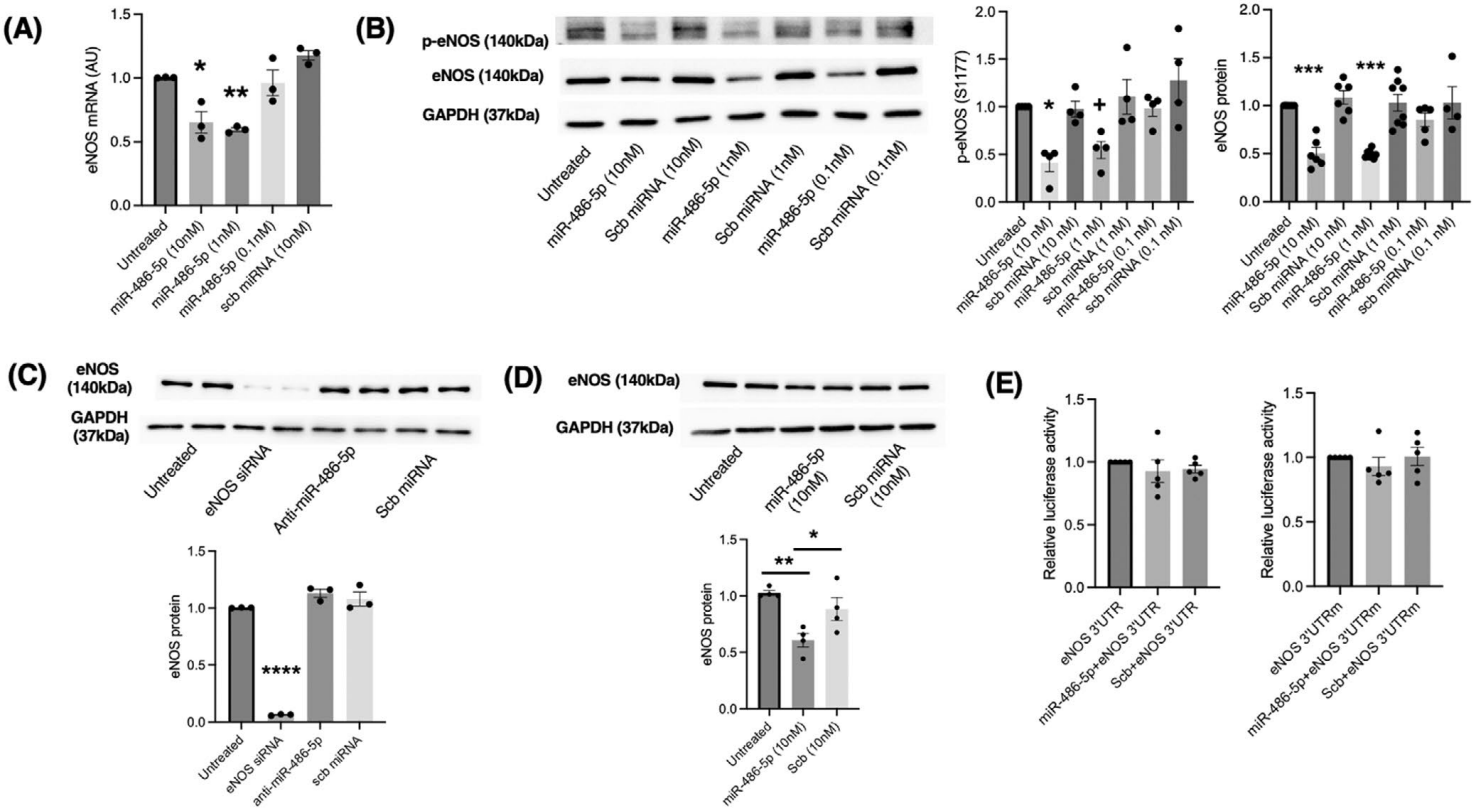
miR‐486‐5p inhibits eNOS mRNA and protein levels but does not target the eNOS 3′ UTR. (A) RT‐qPCR for eNOS mRNA in HUVECs, normalised to GAPDH. **p < 0.01, *p < 0.05 miR‐486‐5p 10 nM and 1 nM vs. untreated, scb miRNA; *n* = 3. (B) eNOS and phospho‐eNOS (S1177) protein levels in HUVECs. Representative immunoblots and densitometry. Protein levels were normalised to GAPDH. ***p < 0.001, miR‐486‐5p 10 nM, 1 nM vs. untreated, scb miRNA (10 nM, 1 nM, 0.1 nM); *p < 0.05, miR‐486‐5p (10 nM) vs. untreated, scb (1 nM, 0.1 nM); ^+^ *p* < 0.05 miR‐486‐5p (1 nM) vs. scb (0.1 nM); *n* = 4–8 experiments. (C) Immunoblot of eNOS protein levels from HUVECs transfected with 5 nM eNOS siRNA, 10 nM miR‐486‐5p antagomir, or 10 nM scb miRNA. eNOS protein levels were normalised to GAPDH. ****p < 0.0001; *n* = 3 experiments. (D) Immunoblot of eNOS protein levels from human pulmonary microvascular endothelial cells (HPMECs) transfected with 10 nM miR‐486‐5p mimic or scb miRNA. eNOS protein levels were normalised to GAPDH. **p < 0.01, *p < 0.05; *n* = 4 experiments. (E) Luciferase reporter assay to assess for miR‐486‐5p binding to the eNOS 3′ UTR (left graph) and a mutated 3′ UTR construct (right graph); *n* = 5 experiments.

A phosphokinase array was first performed to screen for the impact of miR‐486‐5p mimic on protein phosphorylation in HUVECs. miR‐486‐5p decreased the levels of phosphorylated eNOS at serine 1177, but also had an inhibitory effect on the levels of several other phosphorylated kinases (Figure S1). We then tested the effect of miR‐486‐5p transfection on eNOS mRNA and protein in HUVECs. At 1 nM or 10 nM, miR‐486‐5p markedly decreased eNOS mRNA and protein, as well as phospho‐eNOS (S1177) protein levels (Figure 1A,B). We attempted miR‐486‐5p inhibition in HUVECs with miR‐486‐5p antagomir, although the Ct values for endogenous miR‐486‐5p indicated low levels, and a reduction in miR‐486‐5p with miR‐486‐5p antagomir was not demonstrated (data not shown). Furthermore, there was no effect of miR‐486‐5p antagomir on eNOS protein levels, while eNOS silencing confirmed antibody specificity (Figure 1C).

Accordingly, further experiments focussed on a gain‐of‐function approach in endothelial cell functional assays. To determine whether the inhibition of eNOS was specific to HUVECs, we studied the effect of miR‐486‐5p in a different endothelial cell line. In cultured human pulmonary microvascular endothelial cells, miR‐486‐5p also significantly inhibited eNOS protein levels (Figure 1D).

From the miRNA target prediction database (miRDB; https://mirdb.org), eNOS has not been identified as a predicted miR‐486‐5p target. We therefore conducted a luciferase reporter assay, which demonstrated that miR‐486‐5p does not directly target the eNOS 3′ UTR (Figure 1E).

### 3.2 Effect of miR‐486‐5p on HUVEC Proliferation and Migration

**FIGURE 2:**
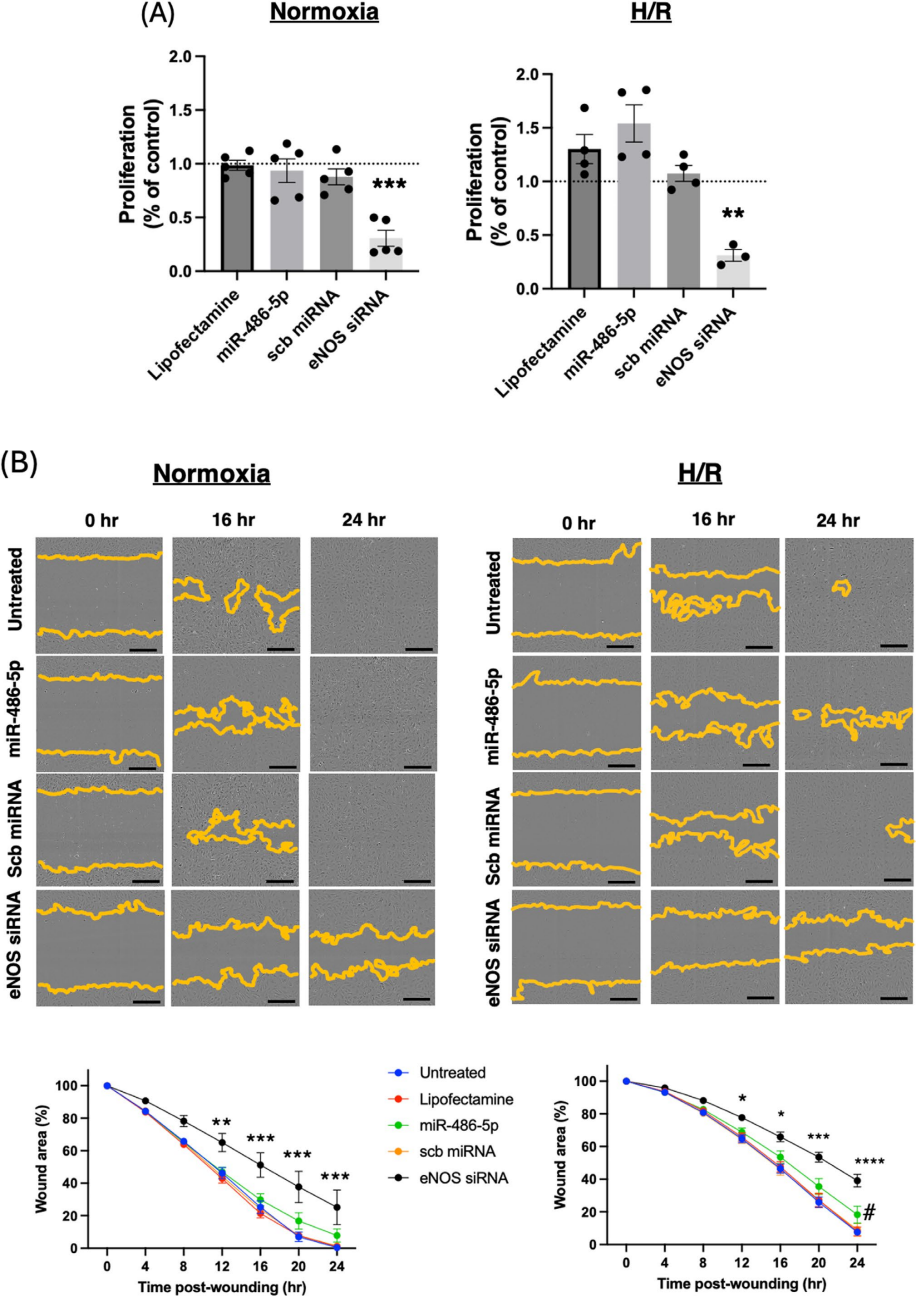
Effect of miR‐486‐5p on cell proliferation and migration in HUVECs under normoxic conditions or subjected to H/R. (A) Cell proliferation was measured by 5‐bromo‐2′‐deoxyuridine (BrdU) assay. ***p < 0.001, **p < 0.01 eNOS siRNA vs. all groups; *n* = 4–5 experiments. (B) Cell migration was evaluated using a scratch wound‐healing assay. Representative images at 0 h, 16 h, and 24 h after wounding are shown, with orange lines indicating the cellular migration front (top). Quantification of wound area closure showed no effect of miR‐486‐5p in normoxia, but a trend towards inhibition of migration by 24 h of re‐oxygenation. *p < 0.05, **p < 0.01, ***p < 0.001, ****p < 0.0001 eNOS siRNA vs. all groups; ^#^ *p* < 0.05, miR‐486‐5p vs. untreated, lipofectamine; *n* = 3–5 experiments. Scale bar = 300 μm.

We evaluated the functional effects of miR‐486‐5p in HUVECs under normoxic conditions and upon exposure to H/R. In either condition, miR‐486‐5p had no impact on cell proliferation, although selective knockdown with eNOS siRNA significantly inhibited this process (Figure 2A). HUVECs subjected to H/R exhibited decreased migration during re‐oxygenation compared to normoxic cells (Figure 2B). Selective eNOS knockdown significantly inhibited cell migration under both conditions (Figure 2B). In contrast, miR‐486‐5p had no impact on cell migration in normoxia but tended to decrease migration by 24 h in H/R (Figure 2B).

### 3.3 Effect of miR‐486‐5p on Network Formation (Angiogenesis)

**FIGURE 3:**
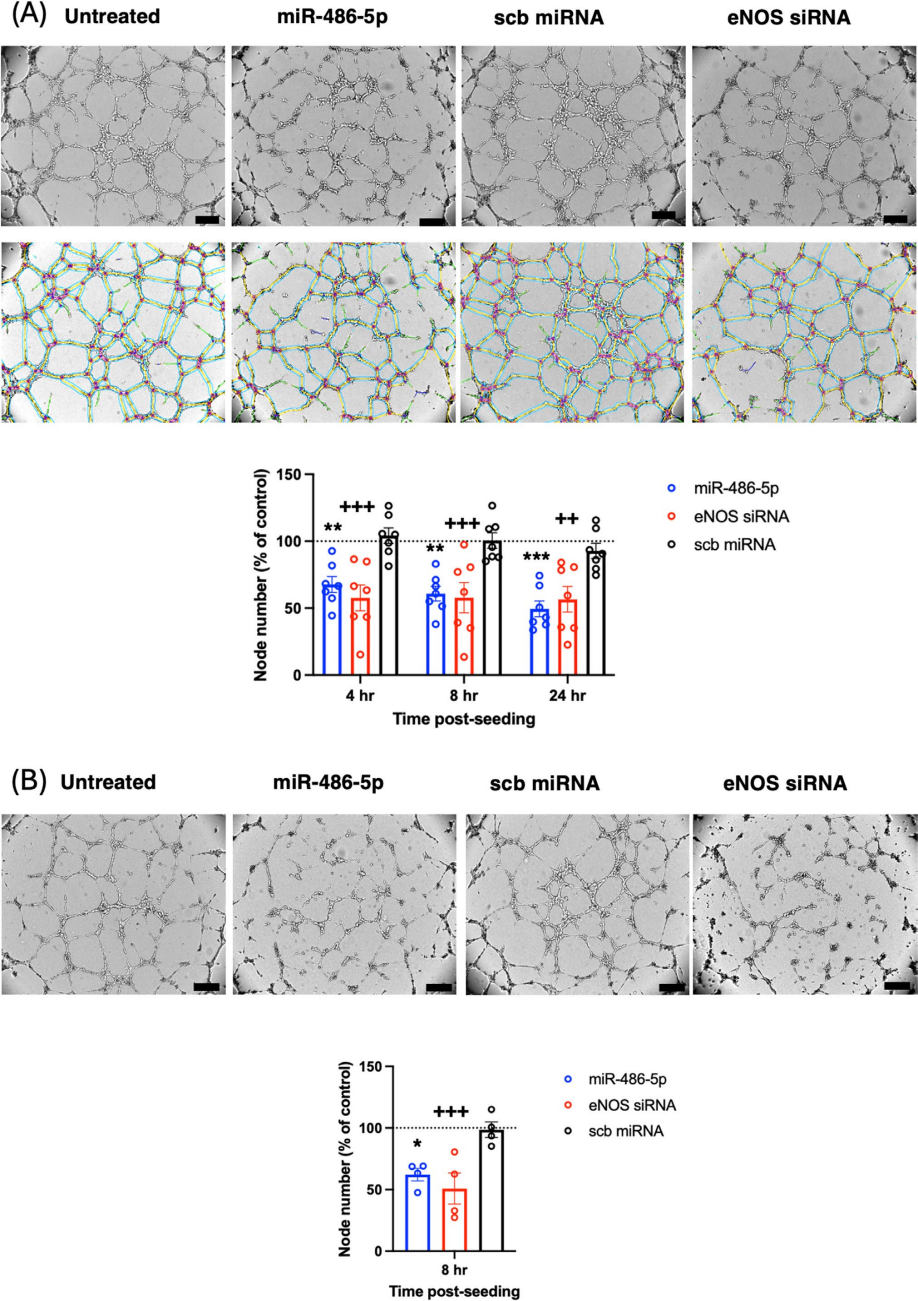
miR‐486‐5p inhibits network formation in HUVECs in (A) normoxia or (B) subjected to H/R. Representative images 8 h after seeding HUVECs in Matrigel, with quantification of network nodes. Representative network traces obtained from Image J are shown (A, bottom). Scale bar = 200 μm. *p < 0.05, **p < 0.01, ***p < 0.001, miR‐486‐5p vs. scb; ^++^ *p* < 0.01, ^+++^ *p* < 0.001, eNOS siRNA vs. scb; *n* = 4–7 experiments (2‐way ANOVA and Tukey's *post hoc* test).

**FIGURE 4:**
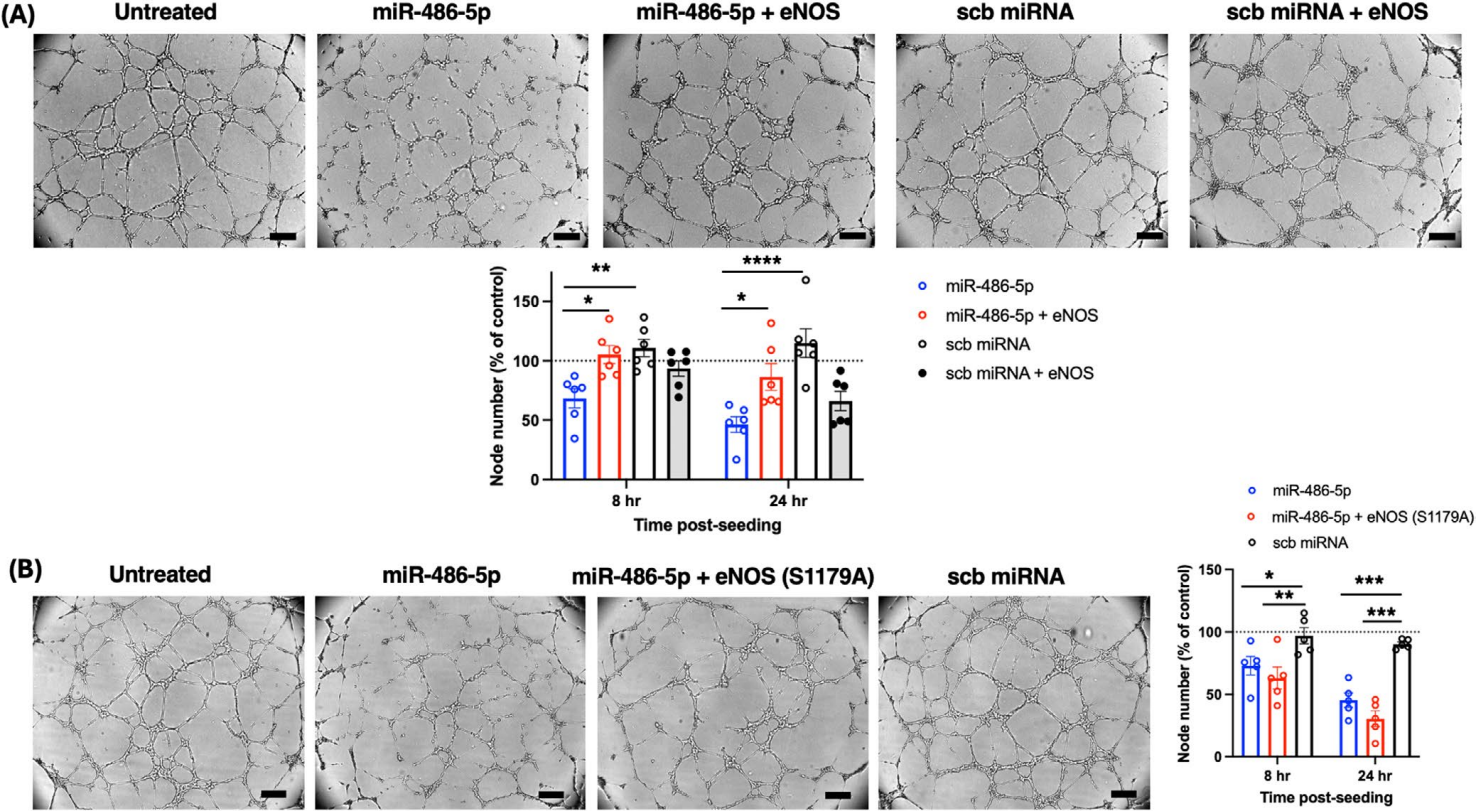
Co‐transfection of HUVECs with miR‐486‐5p and eNOS plasmid restores network formation. (A) Effect of co‐transfection with wild‐type eNOS (*n* = 6) and (B) effect of co‐transfection with mutant inactive eNOS S1179A (*n* = 5). Representative images 8 h after seeding HUVECs in Matrigel, with quantification of network nodes at 8 h and 24 h post seeding. Scale bar = 200 μm; *p < 0.05, **p < 0.01, ***p < 0.001, ****p < 0.0001 (2‐way ANOVA and Tukey's *post hoc* test).

miR‐486‐5p transfection inhibited angiogenesis in normoxic HUVECs, with a decreased number of network nodes at all time points post‐seeding up to 24 h (Figure 3A), and decreased network nodes after 8 h of H/R (Figure 3B). eNOS siRNA transfection had a similar inhibitory effect on angiogenesis in normoxia and with H/R (Figure 3A,B). To determine the impact of miR‐486‐5p‐induced eNOS down‐regulation on HUVEC angiogenesis, we first treated cells with a nitric oxide (NO) donor to attempt to rescue the inhibitory effect of miR‐486‐5p. DETA‐NONOate releases NO at a slow rate and has been shown to induce a pro‐angiogenic response at 10 μM [34]. At a concentration of 10 μM, DETA‐NONOate did not rescue the inhibitory effects of miR‐486‐5p or eNOS siRNA, while at a higher dose (100 μM), it inhibited network formation (data not shown). Consequently, HUVECs were co‐transfected with miR‐486‐5p and eNOS plasmid (to restore eNOS expression) for attempted rescue of impaired angiogenesis. eNOS plasmid transfection increased levels of eNOS protein in these cells (Figure S2). Co‐transfection of eNOS plasmid with miR‐486‐5p restored angiogenesis, with increased network nodes at 8 h and 24 h post seeding (Figure 4A). In contrast, co‐transfection with mutated eNOS at S1179A, lacking the phosphorylation site for activation, did not rescue the anti‐angiogenic effect of miR‐486‐5p (Figure 4B).

### 3.4 Biotinylated miR‐486‐5p Pulldown Identifies miR‐486‐5p Targets in HUVECs

**FIGURE 5:**
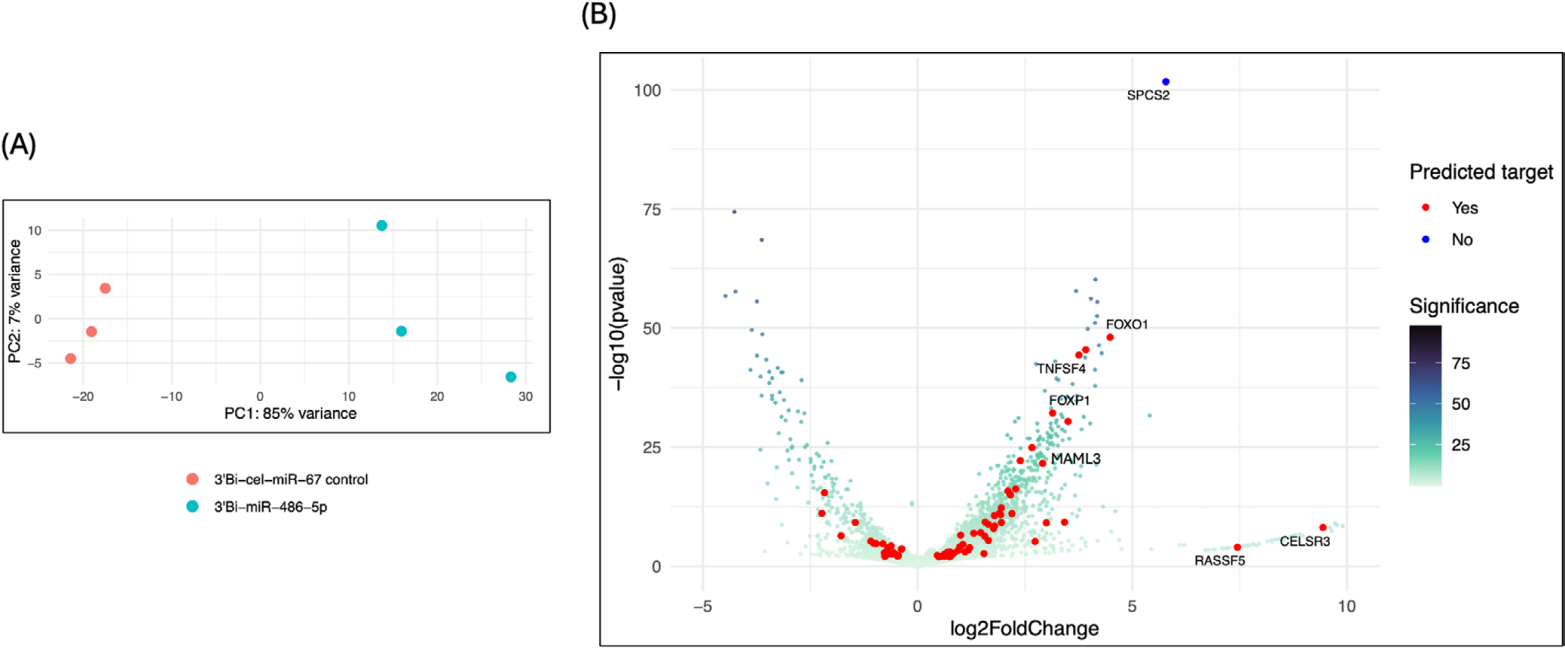
RNA‐sequencing of biotinylated miR‐486‐5p pulldown RNA. (A) Principal component analysis of biotinylated miRNA pulldown RNA‐seq data. The data is segregated between miR‐486‐5p and cel‐miR‐67 negative control pulldown RNA; *n* = 3 samples per condition. (B) Volcano plot of differentially expressed genes in miR‐486‐5p pulldown RNA relative to cel‐miR‐67 control. Differential expression is represented as log_2_ fold change (relative to cel‐miR‐67). The red dots indicate predicted miR‐486‐5p targets from miRDB miRNA target prediction database (https://mirdb.org). All smaller points are significantly differentially expressed genes that are not miRDB predicted miR‐486‐5p targets, coloured according to level of significance. Significance was calculated as –log_10_(*p*adj).

To identify potential targets of miR‐486‐5p that might mediate eNOS inhibition, HUVECs were transfected with biotinylated miR‐486‐5p or biotinylated cel‐miR‐67 negative control for 24 h, followed by biotinylated RNA pulldown with streptavidin beads and RNA sequencing. Initial transcript mapping with filtering to remove genes that were not detected yielded a total of 24,605 genes. Principal component analysis (PCA) revealed a clear separation between the miR‐486‐5p and control RNA pulldowns (Figure 5). After DESEq2 normalisation with a cut‐off adjusted *p* value of 0.01, filtering out genes with low expression levels (7631 genes, 31%) and genes with extreme outlier replicates (126 genes, 0.5%), we retained 2325 significantly differentially expressed genes. Of these, 1729 had higher abundance and 596 lower abundance in the miR‐486‐5p pulldown (File S1).

Comparing RNA‐seq results to predicted miR‐486‐5p targets from miRDB (331 predicted targets), 40 transcripts (12% of predicted targets) were identified as having a fold change significantly different from zero and enriched in the miR‐486‐5p pulldown compared to control. Thus, the majority of genes (1689/1729, 97.7%) identified as enriched in the miR‐486‐5p pulldown are not found in the miRDB predicted target database. From predicted miR‐486‐5p targets, the ones most significantly increased in the miR‐486‐5p pulldown included FOXO1, ZNF37A, TNFSF4, FOXP1, RASSF3, GLIPR2 and GOLGA3, while CELSR3 had the highest fold change (Table 1, Figure 5). Notably, the most statistically significantly enriched transcript from the miR‐486‐5p pulldown RNA was SPCS2, which is not a predicted target of miR‐486‐5p (Figure 5). As expected, eNOS mRNA was not identified in the pulldown.

**TABLE 1:** Most significantly enriched transcripts in miR‐486‐5p pulldown RNA from HUVECs.

Predicted miR‐486‐5p targets			Enriched, not predicted miR‐486‐5p targets		
Gene	Log_2_FC	*p* (adj)	Gene	Log_2_FC	*p* (adj)
FOXO1	4.48	9.65 × 10^−46^	SPCS2	5.78	2.98 × 10^−98^
ZNF37A	3.92	3.41 × 10^−43^	STK39	4.14	2.83 × 10^−57^
TNFSF4	3.76	4.92 × 10^−42^	LSM4	3.69	5.86 × 10^−55^
FOXP1	3.14	7.31 × 10^−30^	MTRES1	4.18	4.94 × 10^−50^
RASSF3	3.50	1.03 × 10^−28^	SP2	4.13	1.28 × 10^−48^
GLIPR2	2.66	2.04 × 10^−23^	H3‐3B	3.96	2.07 × 10^−47^
GOLGA3	2.39	8.47 × 10^−21^	NDEL1	4.22	4.50 × 10^−44^
MAML3	2.91	3.04 × 10^−20^	ETV1	4.29	1.71 × 10^−42^
UBASH3B	2.28	3.87 × 10^−15^	SRC	3.90	1.24 × 10^−41^
FGD6	2.16	5.15 × 10^−14^	FMC1	2.76	2.48 × 10^−40^
NCOA6	2.16	6.19 × 10^−14^	MTF2	4.13	3.61 × 10^−39^
MAVS	1.93	5.31 × 10^−10^	NABP1	3.23	2.07 × 10^−37^
RELT	1.91	2.64 × 10^−10^	RHOBTB2	3.61	2.61 × 10^−36^
ZNF740	3.42	1.52 × 10^−8^	EIF4EBP1	2.96	6.71 × 10^−35^
CELSR3	9.44	1.67 × 10^−7^	ZFP91	3.48	9.60 × 10^−34^

**FIGURE 6:**
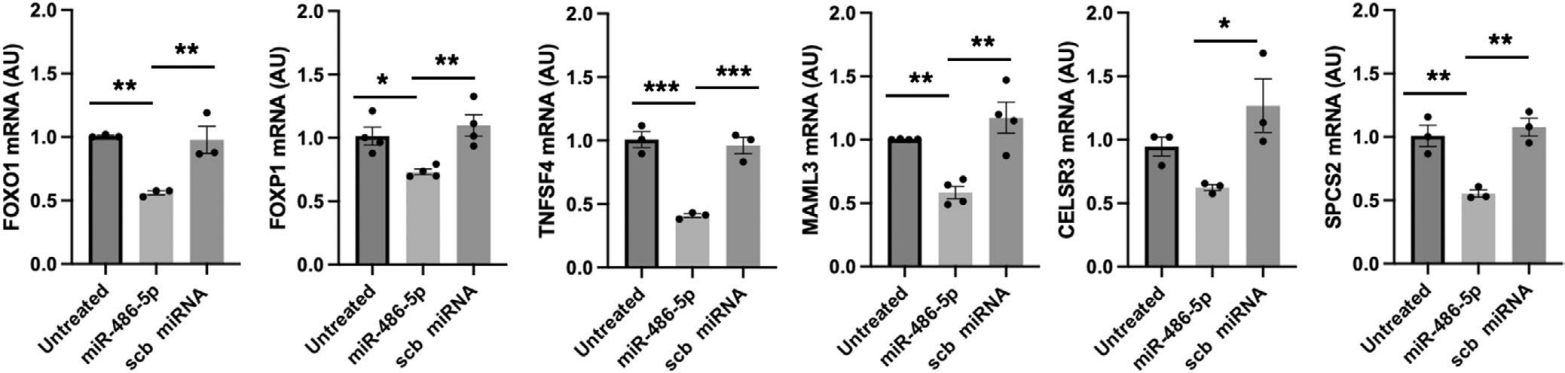
miR‐486‐5p target validation by RT‐qPCR. The levels of 6 enriched transcripts in the pulldown RNA were measured in total RNA from HUVECs transfected with miR‐486‐5p mimic (1 nM) or scb miRNA (1 nM). All 6 transcripts were decreased by miR‐486‐5p. *p < 0.05, **p < 0.01, ***p < 0.001; *n* = 3–4 experiments.

We selected transcripts for validation by qPCR based on their adjusted *p* values and log fold changes. HUVECs were transfected with 1 nM miR‐486‐5p mimic or scb miRNA, and total RNA was isolated after 24 h. FOXO1, FOXP1, TNFS4, MAML3, CELSR3 and SPCS2 mRNAs were significantly decreased by miR‐486‐5p (Figure 6). PTEN mRNA, a validated miR‐486‐5p target reported in several studies including kidney [15] and cardiac I/R injury [11, 35], skeletal muscle disorders [36] and ischaemic stroke [21], was not identified in our miR‐486‐5p pulldown. Although miR‐486‐5p mimic transfection in HUVECs did not decrease PTEN mRNA across a range of concentrations, a small decrease in PTEN protein levels was observed (Figure S3).

### 3.5 Selective Knockdown of MAML3 Inhibits eNOS Protein Expression

**FIGURE 7:**
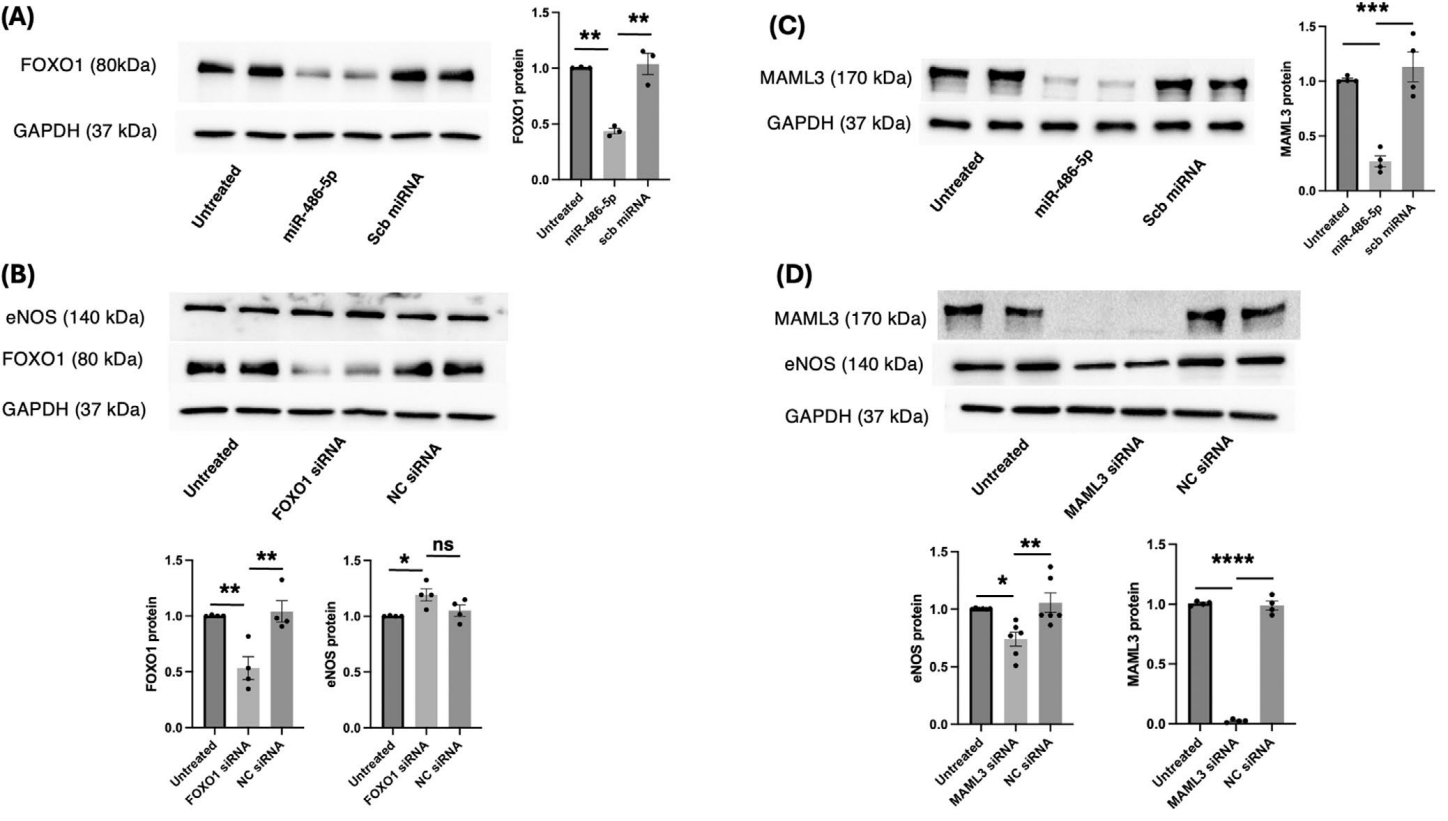
MAML3 silencing decreases eNOS protein levels in HUVECs. (A) Immunoblot demonstrating decreased FOXO1 protein levels by miR‐486‐5p. **p < 0.01; *n* = 3 experiments (B) Selective FOXO1 silencing and eNOS protein levels. *p < 0.05, **p < 0.01, ns = not significant; *n* = 4 experiments (C) Immunoblot demonstrating decreased MAML3 protein levels by miR‐486‐5p. ***p < 0.001; *n* = 4 experiments. (D) Immunoblot of eNOS protein from HUVECs treated with MAML3 siRNA demonstrates decreased eNOS protein levels; *p < 0.05, **p < 0.01; *n* = 6 experiments. MAML3 siRNA also decreased MAML3 protein levels; ****p < 0.0001; n=3 experiments.

We used our miR‐486‐5p pulldown data to probe the link between miR‐486‐5p and eNOS expression. FOXO1 regulates eNOS transcription [37], and FOXO1 protein levels are significantly inhibited by miR‐486‐5p (Figure 7A). siRNA‐mediated FOXO1 knockdown had no statistically significant impact on eNOS protein levels, although there was a trend towards increased eNOS protein (Figure 7B). MAML3 knockdown inhibits angiogenesis linked to miR‐486‐5p [18], and MAML3 is a positive regulator of eNOS activation [38]. We show that miR‐486‐5p significantly decreased MAML3 mRNA (Figure 6) and protein levels (Figure 7C). In contrast to FOXO1, siRNA‐mediated MAML3 knockdown in HUVECs significantly inhibited eNOS protein expression (Figure 7D).

## 4 Discussion

Angiogenesis is the biological process of new blood vessel formation from existing ones [39]. Tight regulation of angiogenesis is critical because while this process is physiological during embryogenesis, wound healing, and I/R injury, it can be pathological in other contexts such as diabetic retinopathy, tumour growth and metastases [39]. In this regard, regulatory miRNA networks play an important role in the temporal coordination of angiogenic processes in endothelial cells, including proliferation and migration [40]. miR‐486‐5p has been reported to have variable effects on angiogenesis, depending on cell type, target organ or direct delivery versus exosomal transfer [18, 19, 20, 21]. On the other hand, eNOS, which generates nitric oxide (NO), is a major regulator of endothelial function [41] and stimulates angiogenesis [42]. Here, we demonstrate that administration of miR‐486‐5p mimic to cultured endothelial cells reduces eNOS protein expression and inhibits angiogenesis. Inhibition of eNOS expression was observed in two cell lines, suggesting conservation of effect across human endothelial cell types. The inhibitory effect of miR‐486‐5p on angiogenesis was rescued by co‐transfecting eNOS plasmid. Although our data cannot completely exclude an effect of miR‐486‐5p on kinases targeting eNOS phosphorylation (S1177), our phosphokinase array revealed no significant differences in the levels of phosphorylated Akt (S473, T308) or in checkpoint kinase (Chk‐2) by miR‐486‐5p, and kinases targeting eNOS phosphorylation (S1177) were not enriched in the miR‐486‐5p pulldown RNA. Taken together, these data suggest that the anti‐angiogenic effect of miR‐486‐5p is mediated through inhibition of eNOS expression. Finally, since eNOS mRNA is not a direct target of miR‐486‐5p, biotinylated miRNA pulldown of transcripts was conducted to identify a potential pathway regulating eNOS expression. We identified MAML3 as a highly enriched target of miR‐486‐5p, and its silencing significantly inhibited eNOS protein expression. These data suggest a pathway whereby miR‐486‐5p targets MAML3 mRNA, leading to inhibition of eNOS expression and reduction in angiogenesis.

MAML3 is a nuclear protein that functions as a transcriptional co‐activator for Notch signalling, a highly conserved pathway that influences cell fate decisions [43], organogenesis, and tumour angiogenesis [38]. Notch signalling activates eNOS during embryonic cardiac development [44]. In a mouse model, Patenaude et al. demonstrated that endothelial Notch was required for tumour growth and perfusion in response to vascular endothelial growth factor (VEGF) by regulating eNOS activation [38]. Rosano et al. showed that long non‐coding RNA LINC02802 promotes sprouting angiogenesis in HUVECs by acting as a competing endogenous RNA for miR‐486‐5p [18]. Furthermore, overexpression of MAML3 counter‐acted miR‐486‐5p's inhibitory effect, supporting a role for Notch signalling in angiogenesis [18]. Our data in HUVECs are in concordance with Rosano et al. [18] although the mechanism by which miR‐486‐5p targeting of endothelial MAML3 regulates eNOS expression and NOTCH signalling requires further study.

Unexpectedly, we found that miR‐486‐5p had no significant effect on endothelial cell migration or proliferation while selective eNOS knockdown inhibited both processes. Although we focused on MAML3, individual miRNAs have numerous gene targets. For example, we validated that miR‐486‐5p decreased FOXO1 mRNA and protein levels, but also showed that silencing of FOXO1 had no statistically significant impact on eNOS protein levels, although there was a trend towards increased eNOS protein levels. FOXO1 is a transcription factor involved in the regulation of cell proliferation, apoptosis, and metabolism [45]. Endothelial‐specific deletion of FOXO1 in mice increased endothelial cell proliferation whereas overexpression restricted vascular expansion, thus implicating FOXO1 as a regulator of vascular growth [46]. In addition, a study of postnatal neovascularisation demonstrated that FOXO1 bound the eNOS promoter, inhibited eNOS protein expression, and inhibited angiogenesis in cultured endothelial cells [47]. Thus, targeting of FOXO1 by miR‐486‐5p could plausibly compensate for the functional consequences of miR‐486‐5p‐mediated eNOS inhibition, although the effect of FOXO1 on NO production remains unknown. Consequently, the absence of effect of miR‐486‐5p on endothelial cell proliferation or migration could be due to targeting of FOXO1 (or other targets) involved in these cellular processes.

Several techniques are available to probe for miRNA–mRNA interactions. High‐throughput sequencing of RNAs isolated by crosslinking immunoprecipitation (HITS‐CLIP) [48] and photoactivatable‐ribonucleoside‐enhanced crosslinking and immunoprecipitation (PAR‐CLIP) [4] identify mRNA fragments bound to Argonaute proteins, but infer interactions between protein‐bound mRNAs and miRNAs bioinformatically [6]. In contrast, the method used here, involving transfection of biotinylated miRNA followed by streptavidin purification and RNA sequencing of bound mRNA, identifies transcripts with a high degree of specificity [49, 50]. The number of transcripts enriched in our pulldown RNA is consistent with other studies that used either biotinylated miRNA pulldown [6] or other target identification approaches [4]. For instance, Martin et al. used the biotin pulldown technique on 10 miRNAs, identifying an average of 1500 genes per miRNA that were significantly enriched in the pulldown RNA, with the majority as non‐predicted targets [6].

False‐positive targets could arise from non‐specific interactions, but the complexity of miRNA–mRNA interactions also provides a plausible explanation for the large number of transcripts enriched in the miR‐486‐5p pulldown RNA, exceeding the predicted targets from miRDB (331 transcripts, 40 of which were identified in our pulldown). Similarly, a study by Tan et al. used biotinylated miR‐522 pulldown and identified 547 enriched transcripts, of which only 53 were predicted by TargetScan [49]. Although canonical binding involves the miRNA seed in its 5′ region (nucleotides 2–7), the presence of imperfect seed matches has been reported [51]. Other miRNA‐responsive elements (MREs) for miRNA/mRNA pairing include miRNA‐centred sites [52], and extended base pairing within the 3′ region of miRNA, known as 3′ supplemental binding [53] or 3′ compensatory binding [2]. MREs have also been identified within 5′ UTRs [3] and within coding regions [3, 54]. In this regard, Martin et al. showed that 82.7% of their significantly enriched transcripts contained a seed or centered miRNA binding site, suggesting that this approach identifies miRNA targets with high sensitivity while minimising false positive target identification [6]. In the current studies, we identified SPCS2 as a highly enriched target, although it is absent from miR‐486‐5p target prediction databases. However, manual analysis of the SPCS2 3′ UTR sequence (UCSC Genome Browser, https://genome.ucsc.edu) reveals that it contains a binding site to the miR‐486‐5p seed region (position 2–7 from the 5′ end of miR‐486‐5p). Consequently, our miR‐486‐5p pulldown RNA‐seq data are likely to include novel miR‐486‐5p targets in cultured endothelial cells.

We acknowledge certain limitations of our study due to cell model and other methodologies. HUVECs are macrovascular endothelial cells and are therefore not the ideal model to recapitulate microcirculatory pathophysiology [55] and our study provides limited data on pulmonary microvascular endothelial cells. Biotinylated miRNA pulldown can detect targets that are translationally repressed, but is less likely to detect targets that are degraded [6]. In this regard, inhibition of PTEN protein by miR‐486‐5p has been reported in cardiomyocytes [11], skeletal muscle [36], brain microvascular endothelial cells [21], and in HUVECs subjected to H/R and treated with exosomes enriched in miR‐486‐5p [15]. However, PTEN was not enriched in our pulldown RNA. Given there was a trend for decreased PTEN protein levels by miR‐486‐5p, transcript degradation remains a possibility. Other considerations include the effects of cell type [7, 56] and cellular environment [57] on miRNA–target interactions and cellular functions. In addition, changes in mRNA or protein levels also depend on rates of transcription [58] and protein stability or half‐life [59]. Finally, administration of exogenous miRNA mimic in vitro could account for reported differences in miR‐486‐5p targets and functions. For example, only exosomal miR‐486‐5p confers pro‐angiogenic effects across diverse experimental models [19, 20, 21], in contrast to the inhibitory effects of miR‐486‐5p mimic in the present studies, suggesting other cargo within exosomes such as the RNA‐induced silencing complex (RISC) along with the transferred miRNA. Transfection of exogenous miRNA mimic in vitro delivers supraphysiological levels that compete for the RISC, reducing the availability of miRNA binding proteins for endogenous miRNAs [57]. Consequently, administration of exogenous miRNAs may reduce the effectiveness of endogenous miRNA target gene repression [60].

In summary, we show that miR‐486‐5p inhibits angiogenesis in HUVECs, mediated by down‐regulation of eNOS expression. Biotinylated miR‐486‐5p pulldown identified an enrichment of multiple transcripts in HUVECs, reflecting both predicted and novel targets. Targeting of MAML3 by miR‐486‐5p contributes to the inhibition of eNOS expression and thereby angiogenesis, implicating the Notch pathway as a regulator of eNOS expression and endothelial cell function.

## Author Contributions

**Adrianna Douvris:** conceptualization (supporting), data curation (supporting), formal analysis (lead), investigation (lead), methodology (equal), project administration (supporting), visualization (lead), writing – original draft (lead), writing – review and editing (lead). **Ali Maadelat:** formal analysis (supporting), investigation (supporting), writing – review and editing (supporting). **Christopher J. Porter:** data curation (lead), formal analysis (supporting), methodology (supporting), resources (supporting), software (lead), visualization (supporting), writing – review and editing (supporting). **Dylan Burger:** resources (supporting), supervision (supporting), writing – review and editing (supporting). **Kevin D. Burns:** conceptualization (lead), funding acquisition (lead), methodology (equal), project administration (lead), resources (lead), supervision (lead), writing – review and editing (supporting).

## Conflicts of Interest

The authors declare no conflicts of interest.

## Supporting information


Appendix S1

## Data Availability

The data set from the biotinylated miR‐486‐5p pulldown RNA sequencing is available from the GEO repository (GSE281065). All data are available by contacting the corresponding author.
