# Supplementary material for: miR‐486‐5p Inhibits eNOS and Angiogenesis in Cultured Endothelial Cells by Targeting MAML3
Source: J Cell Mol Med. 2025 May 27;29(11):e70589. doi: 10.1111/jcmm.70589 (PMC12116925; doi:10.1111/jcmm.70589)
Supplement: Supplementary file 1 — Appendix S1 [file JCMM-29-e70589-s001.zip › Captions for supplementary material.docx]

**Supplemental figure 1.** Phosphokinase antibody array from HUVECs transfected with miR-486-5p mimic or scb miRNA. **Left:** images of membranes (phospho-proteins are in duplicate). **Right:** Array legend for coordinates, protein target and phosphorylation site

**Supplemental figure 2.** **eNOS plasmid transfection in HUVECs.** HUVECs were reverse-transfected with eNOS plasmid, miR-486-5p mimic alone, or eNOS plasmid with miR-486-5p mimic. HUVECs were lysed after 48 hr for immunoblot. eNOS protein levels were normalized to GAPDH. *p<0.05 (n=3 experiments)

**Supplemental figure 3. Effect of miR-486-5p on PTEN mRNA and protein levels in HUVECs.** HUVECs were transfected with miR-486-5p mimic or scb miRNA at a range of concentrations (0.1 to 10 nM). (A) PTEN mRNA measured 24 hr post-transfection; n=3 experiments. (B) PTEN protein was evaluated by immunoblot 48 hr post-transfection. PTEN protein levels were normalized to GAPDH. Note that miR-486-5p (5 nM) is not depicted in the densitometry graph because this dose was only used once.
*p<0.05 miR-486-5p (1 nM, 0.5 nM) vs untreated, scb miRNA (1 nM); n=3-7 experiments

**Supplementary File 1.** miR-486-5p mRNA pulldown data

**Supplementary File Western Blots.** Original western blot images
