# Supplementary material for: miR‐486‐5p Inhibits eNOS and Angiogenesis in Cultured Endothelial Cells by Targeting MAML3
Source: J Cell Mol Med. 2025 May 27;29(11):e70589. doi: 10.1111/jcmm.70589 (PMC12116925; doi:10.1111/jcmm.70589)
Supplement: Supplementary file 1 — Appendix S1 [file JCMM-29-e70589-s001.zip › jcmm70589-sup-0001-Figures.pdf]

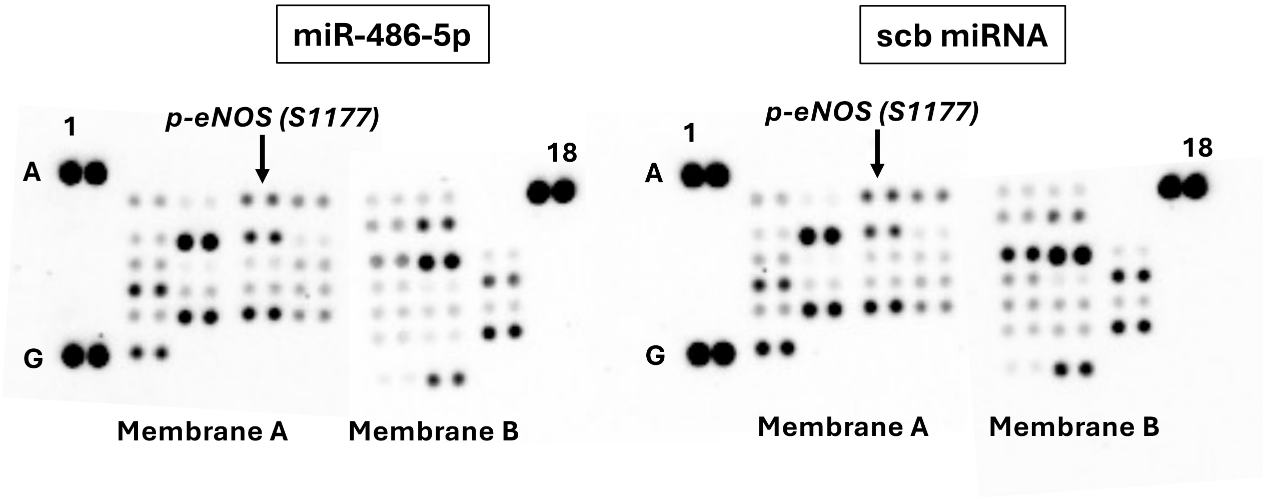

Phosphokinase Array Legend

Spot	Protein	Phospho-site
**A**-A1,2	Reference spot	---
**B**-A11,12	Akt 1/2/3	T308
**B**-A13,14	Akt 1/2/3	S473
**B**-A17,18	Reference spot	---
**A**-B3,4	CREB	S133
**A**-B5,6	EGFR	Y1086
**A**-B7,8	eNOS	S1177
**A**-B9,10	ERK1/2	T202/Y204 T185/Y187
**B**-B11,12	Chk-2	T68
**B**-B13,14	c-Jun	S63
**A**-C3,4	Fgr	Y412
**A**-C5,6	GSK-3α/ß	S21/S9
**A**-C7,8	GSK-3ß	S9
**A**-C9,10	HSP27	S78/S82
**B**-C11,12	p53	S15
**B**-C13,14	p53	S46
**B**-C15,16	p53	S392
**A**-D3,4	JNK1/2/3	T183/Y185/ T221/Y223
**A**-D5,6	Lck	Y394
**A**-D7,8	Lyn	Y397
**A**-D9,10	MSK1/2	S376/S360
**B**-D11,12	P70 S6 kinase	T389
**B**-D13,14	P70 S6 kinase	T421/S424
**B**-D15,16	PRAS40	T246
**A**-E3,4	p38α	T180/Y182
**A**-E5,6	PDGFRß	Y751
**A**-E7,8	PLC-∂1	Y783
**A**-E9,10	Src	Y419
**B**-E11,12	PYK2	Y402
**B**-E13,14	RSK1/2	S221/S227
**B**-E15,16	RSK1/2/3	S380/S386/ S377
**A**-F3,4	STAT2	Y689
**A**-F5,6	STAT5a/b	Y694/Y699
**A**-F7,8	WNK1	T60
**A**-F9,10	Yes	Y426
**B**-F11,12	STAT1	Y701
**B**-F13,14	STAT3	Y705
**B**-F15,16	STAT3	S727
**A**-G1,2	Reference spot	---
**A**-G3,4	ß-catenin	---
**A**-G9,10 **B**-G17,18	PBS (negative control)	---
**B**-G11,12	STAT6	Y641
**B**-G13,14	HSP60	---

Supplemental figure 1. Phosphokinase antibody array from HUVECs transfected with miR-486-5p mimic or scb miRNA.
Left: images of membranes (phospho-proteins are in duplicate).
Right: Array legend for coordinates, protein target and phosphorylation site

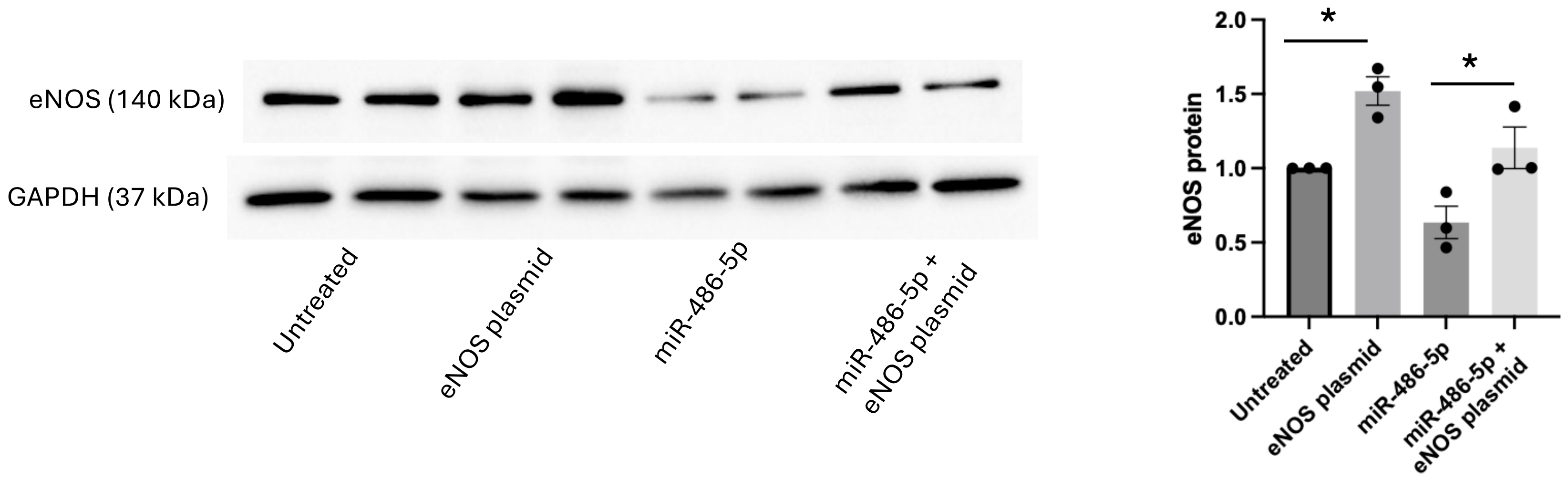

Supplemental figure 2. eNOS plasmid transfection in HUVECs. HUVECs were reverse-transfected with eNOS plasmid, miR-486-5p mimic alone, or eNOS plasmid with miR-486-5p mimic. HUVECs were lysed after 48 hr for immunoblot. eNOS protein levels were normalized to GAPDH. *$p<0.05$ (n=3 experiments)

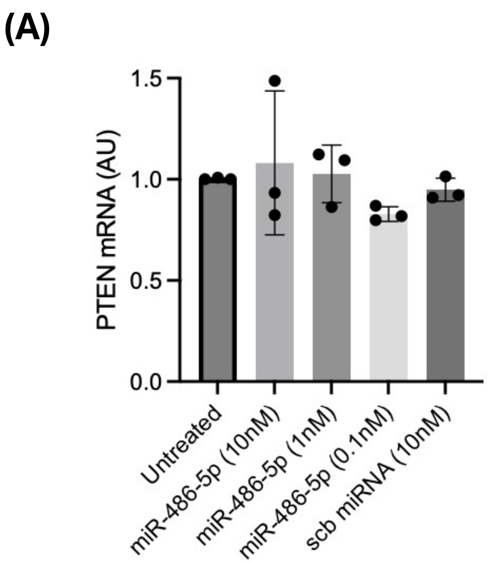

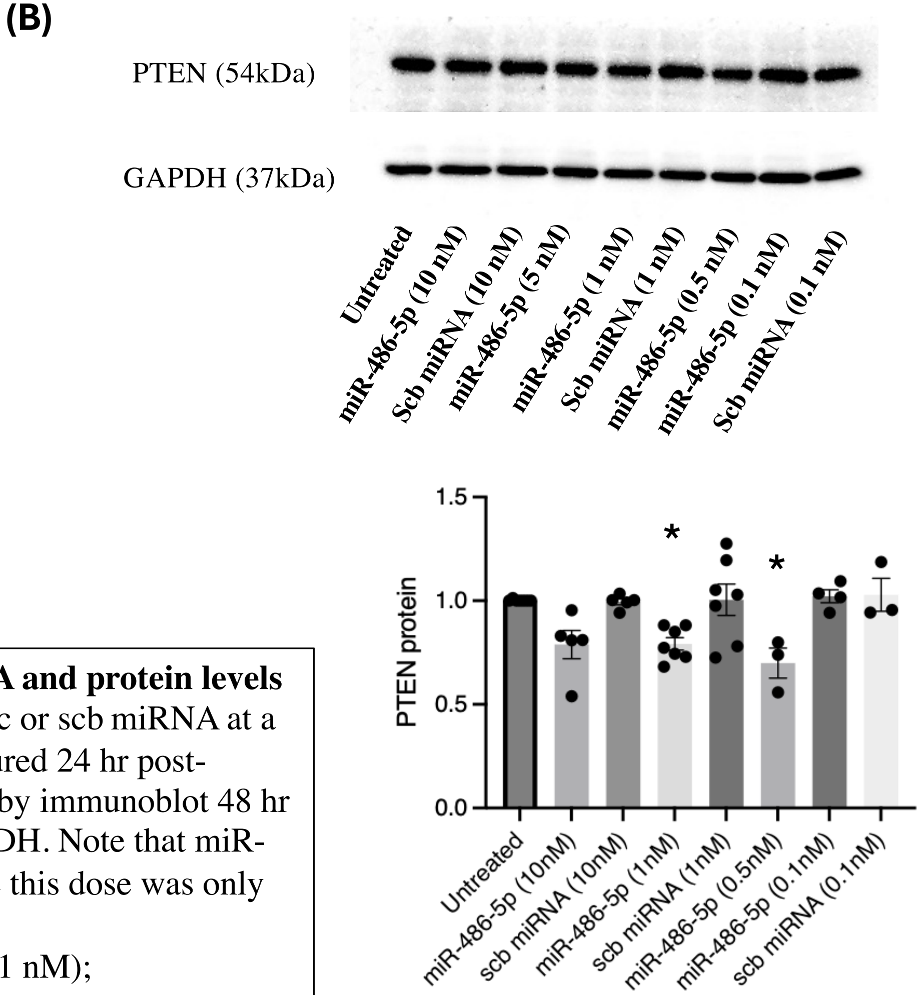

Supplemental figure 3. Effect of miR-486-5p on PTEN mRNA and protein levels in HUVECs. HUVECs were transfected with miR-486-5p mimic or scb miRNA at a range of concentrations (0.1 to 10 nM). (A) PTEN mRNA measured 24 hr post-transfection; n=3 experiments. (B) PTEN protein was evaluated by immunoblot 48 hr post-transfection. PTEN protein levels were normalized to GAPDH. Note that miR-486-5p (5 nM) is not depicted in the densitometry graph because this dose was only used once.
*p<0.05 miR-486-5p (1 nM, 0.5 nM) vs untreated, scb miRNA (1 nM);
n=3-7 experiments
