# Supplementary material for: miR‐486‐5p Inhibits eNOS and Angiogenesis in Cultured Endothelial Cells by Targeting MAML3
Source: J Cell Mol Med. 2025 May 27;29(11):e70589. doi: 10.1111/jcmm.70589 (PMC12116925; doi:10.1111/jcmm.70589)
Supplement: Supplementary file 1 — Appendix S1 [file JCMM-29-e70589-s001.zip › jcmm70589-sup-0003-FileS2.pdf]

Rep#1

eNOS (140kDa)

Figure 1B
HUVEC

PTEN (54kDa)

Supp Fig. 3

GAPDH (37kDa)

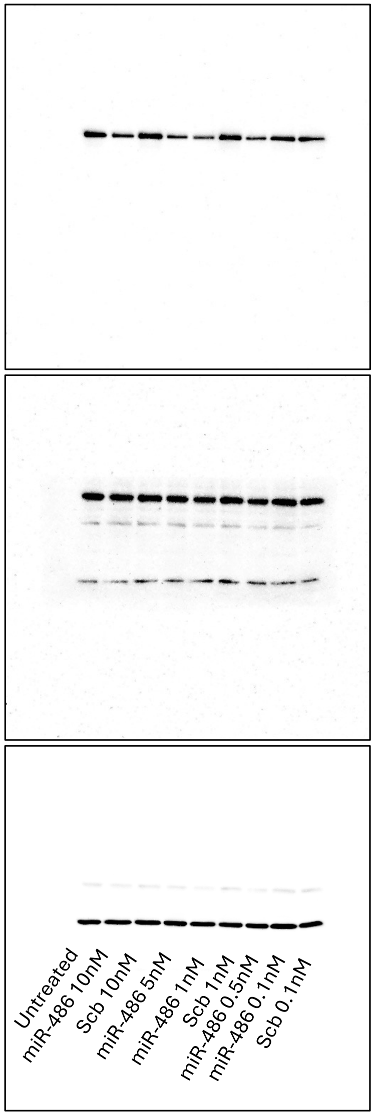

Rep#2

eNOS (140kDa)

Figure 1B
HUVEC

PTEN (54kDa)

GAPDH (37kDa)

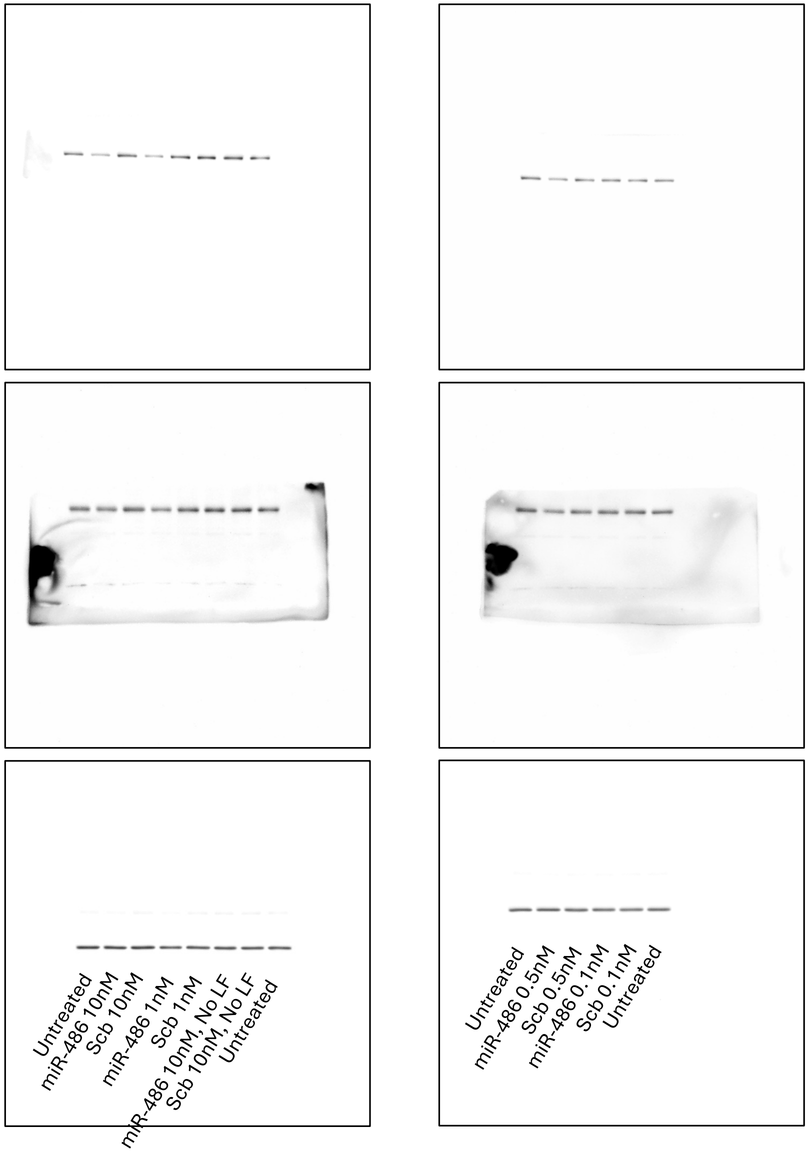

Rep#3

eNOS (140kDa)

Figure 1B
HUVEC

PTEN (54kDa)

Supp Fig. 3

GAPDH (37kDa)

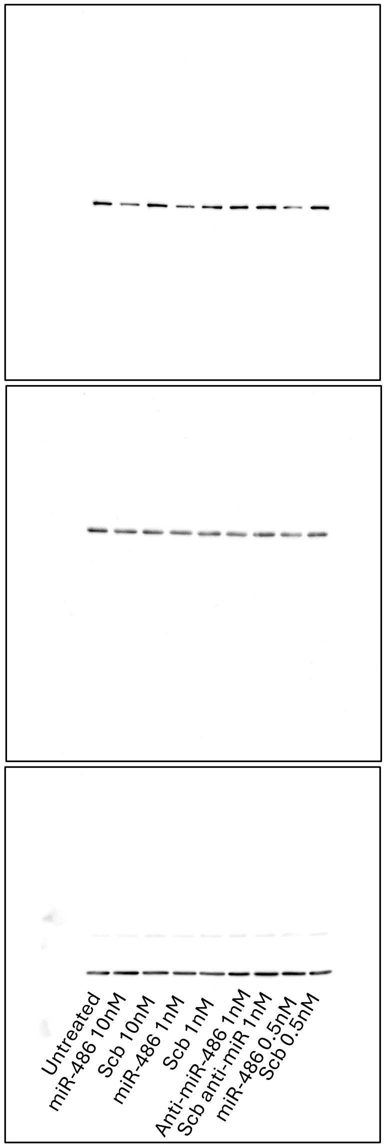

Rep#4

eNOS (140kDa)

Figure 1B
HUVEC

PTEN (54kDa)

Supp Fig. 3

GAPDH (37kDa)

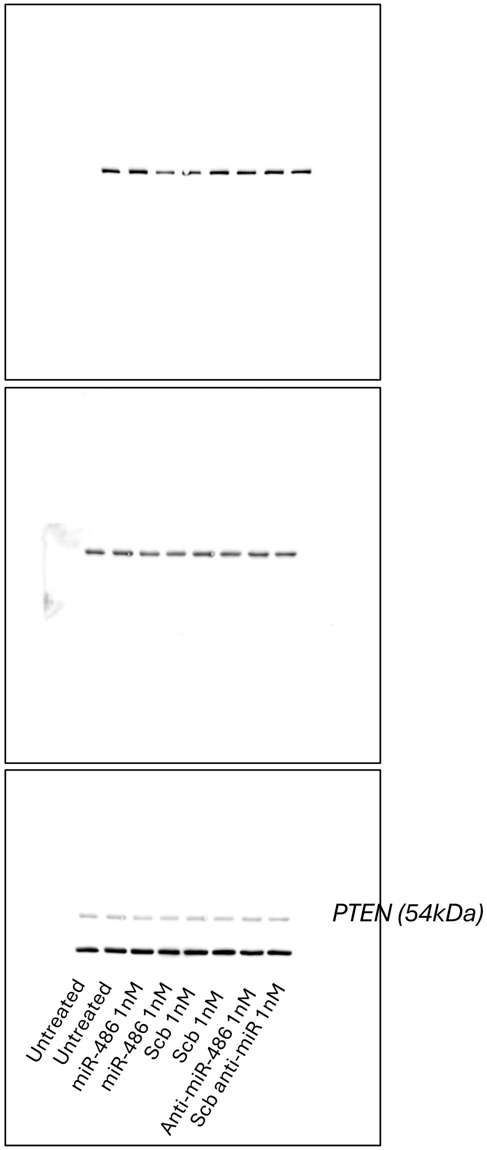

PTEN (54kDa)

Rep#5

eNOS (140kDa)

Figure 1B
HUVEC

PTEN (54kDa)

Supp Fig. 3

GAPDH (37kDa)

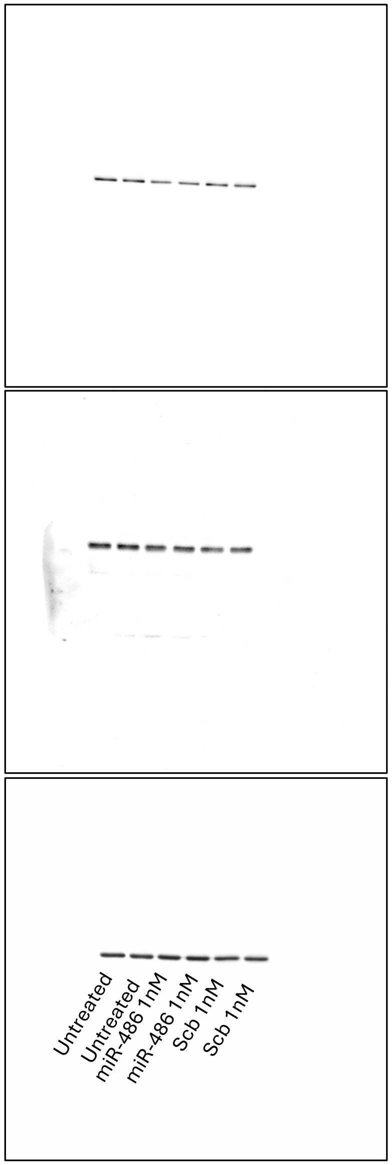

Rep#6

eNOS (140kDa)

Figure 1B
HUVEC

PTEN (54kDa)

Supp Fig. 3

GAPDH (37kDa)

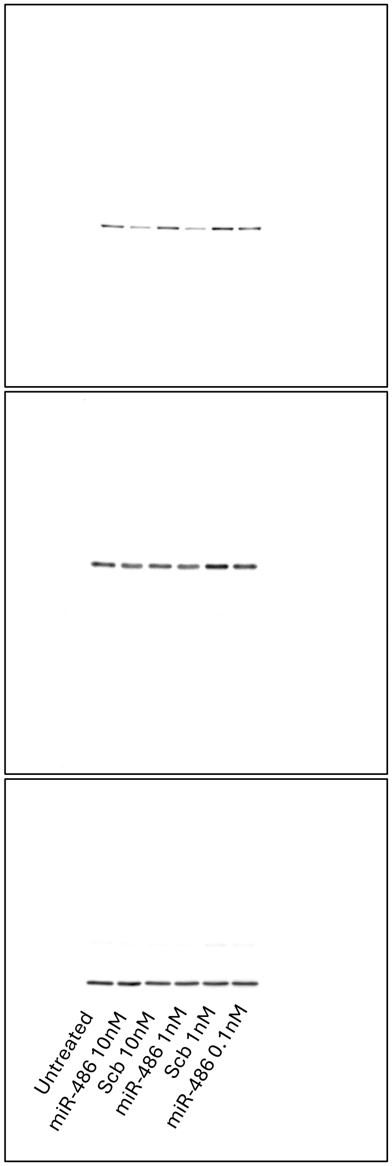

Rep#7

Figure 1B
HUVEC

Supp Fig. 3

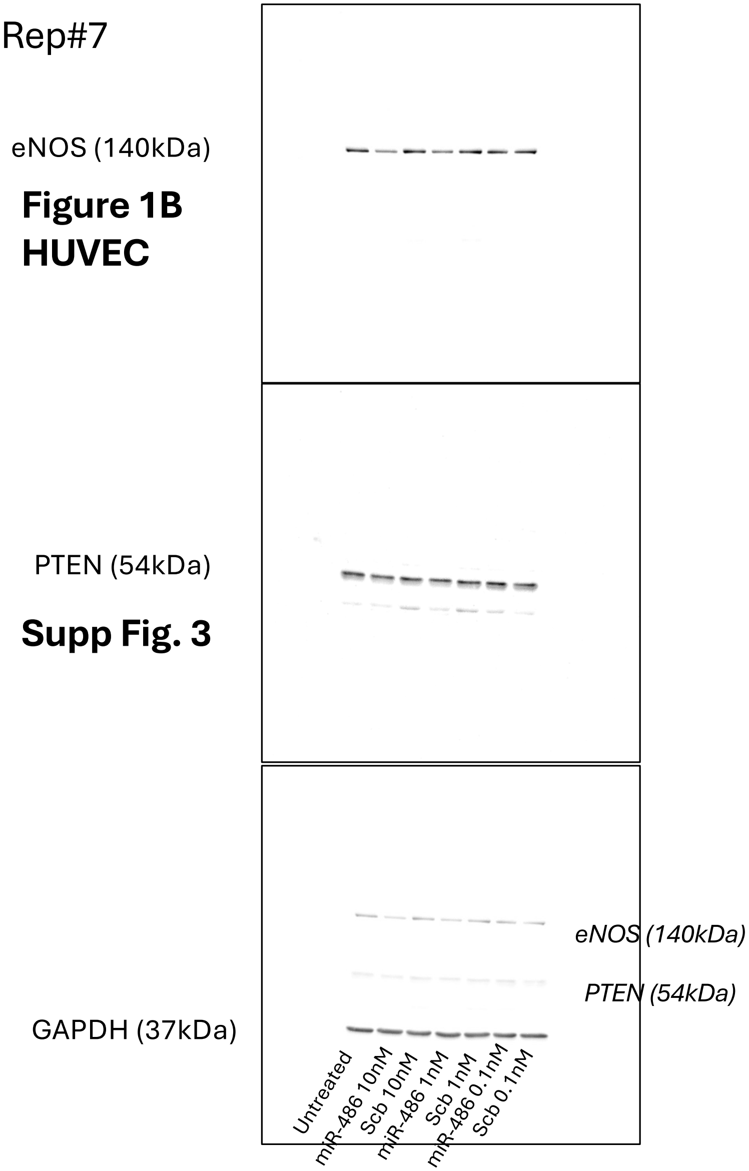

Rep#8

Figure 1B: eNOS (HUVEC)

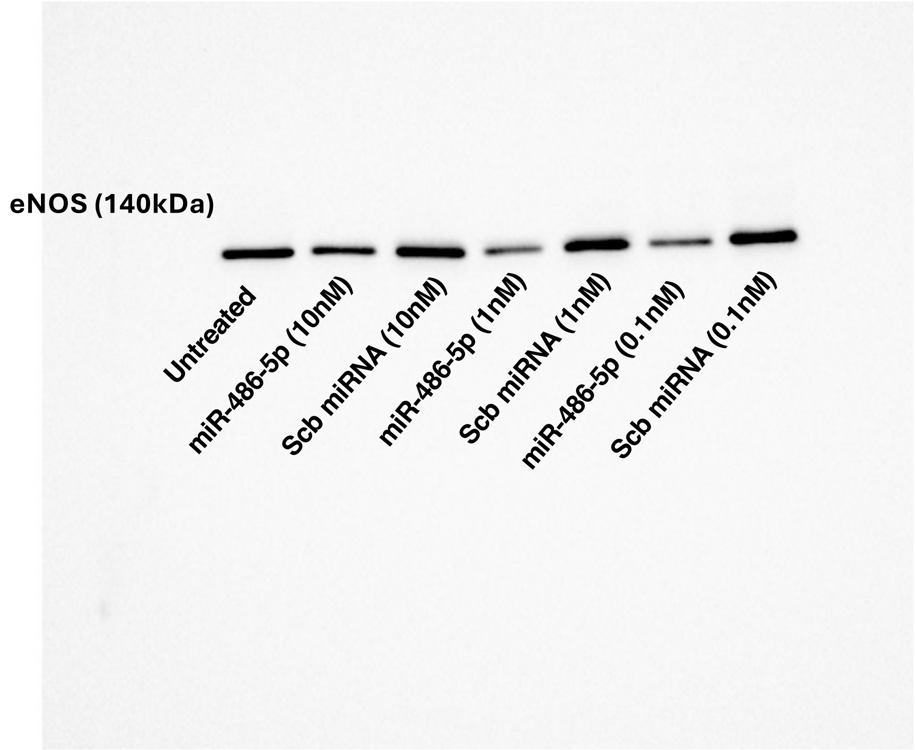

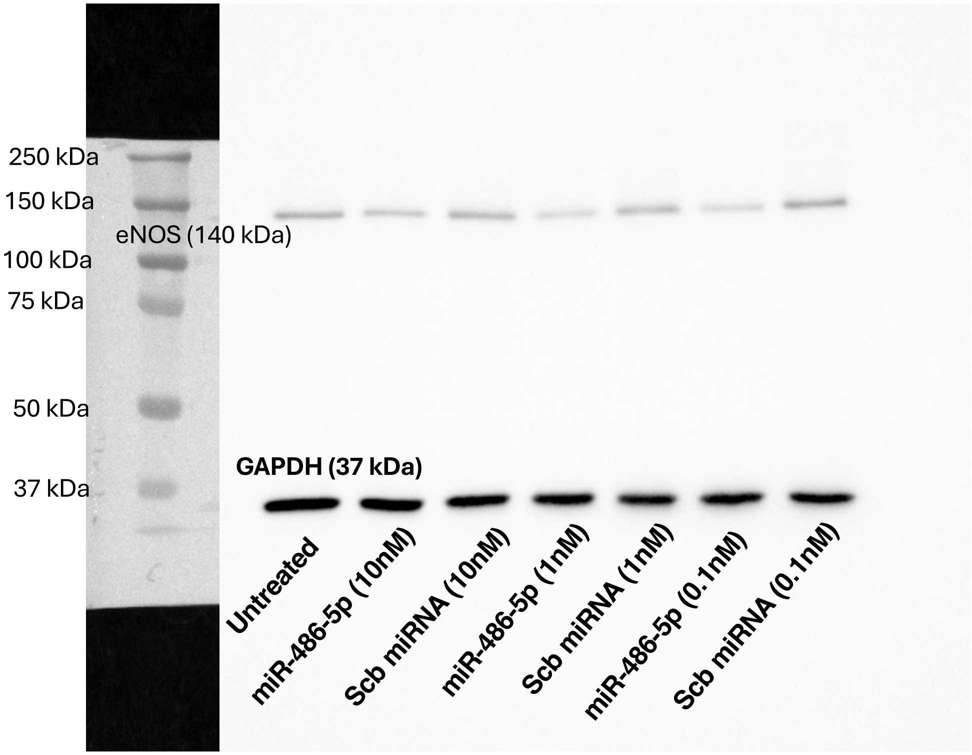

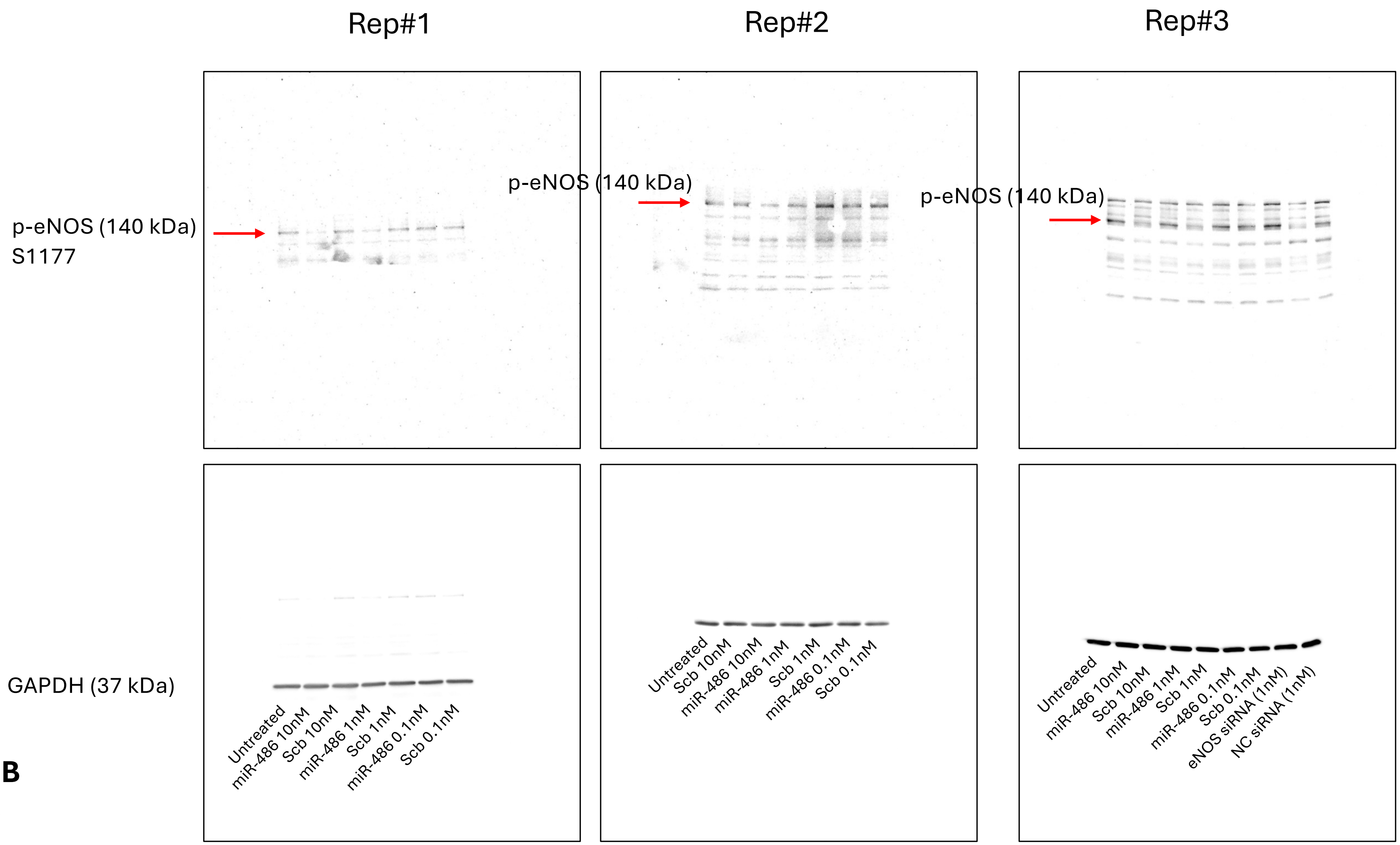

Figure 1B
p-eNOS
HUVEC

Rep#4

Figure 1B: p-eNOS (S1177) (HUVEC)

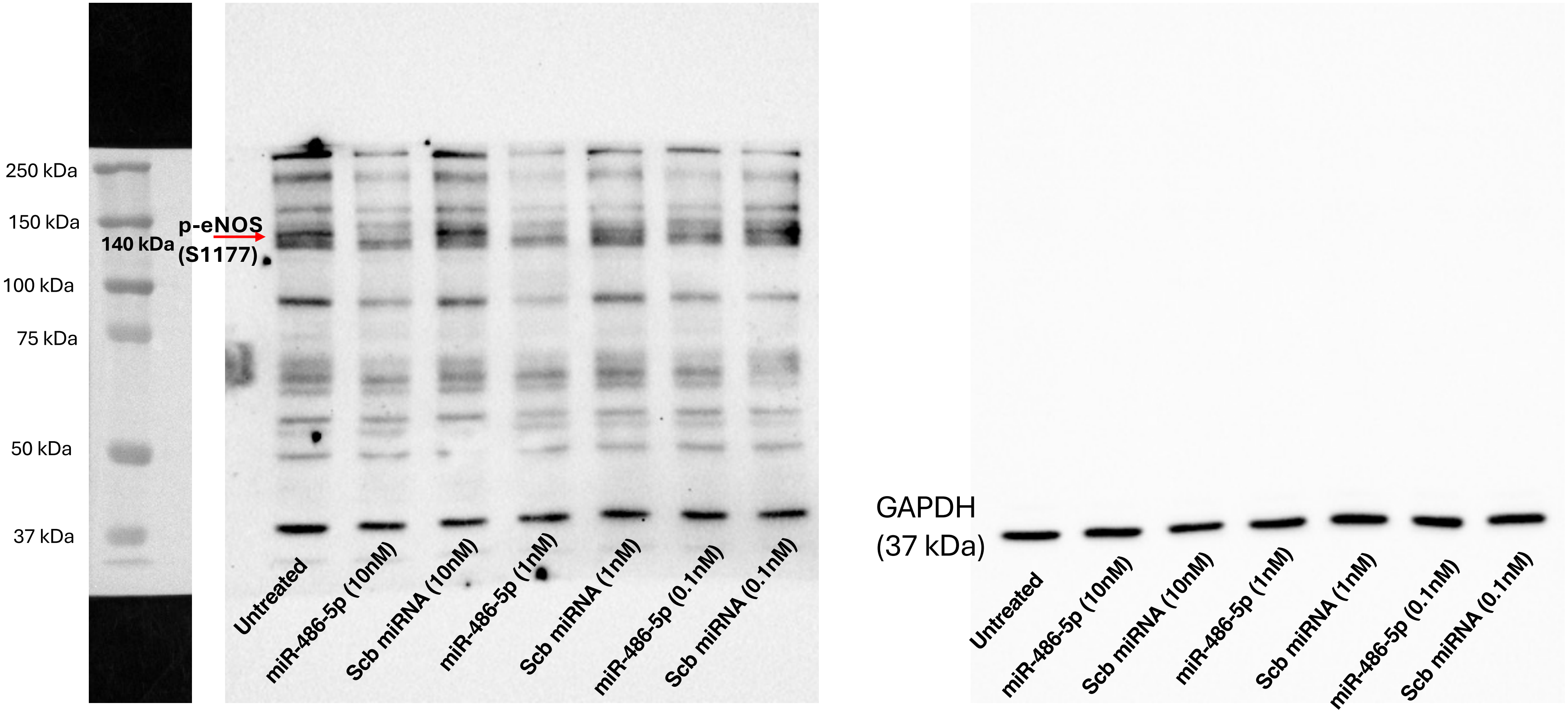

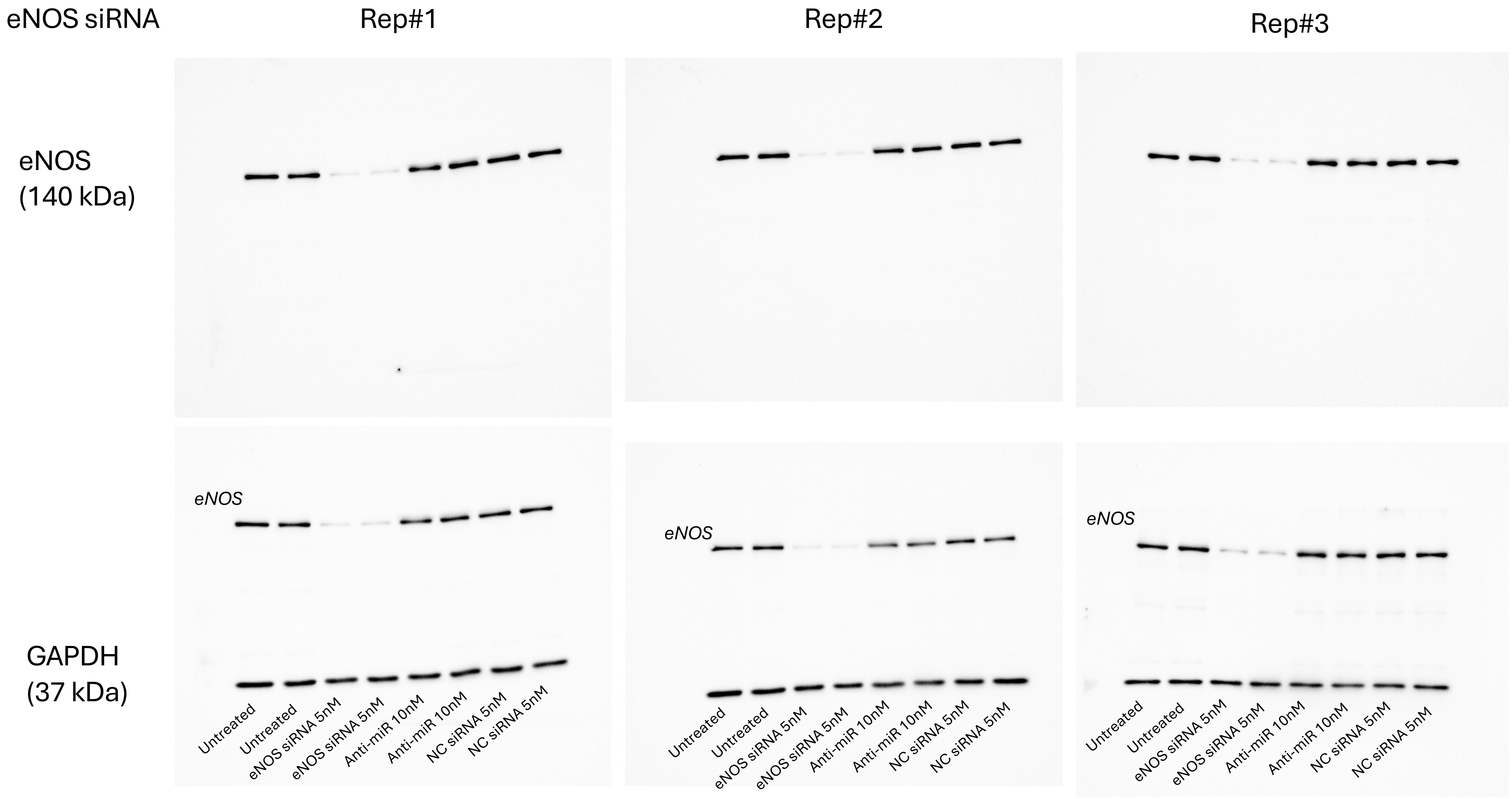
eNOS siRNA
Rep#1
Rep#2
Rep#3
eNOS
(140 kDa)
GAPDH
(37 kDa)
eNOS
Untreated
Untreated
eNOS siRNA 5nM
eNOS siRNA 5nM
Anti-miR 10nM
Anti-miR 10nM
NC siRNA 5nM
NC siRNA 5nM

Figure 1C

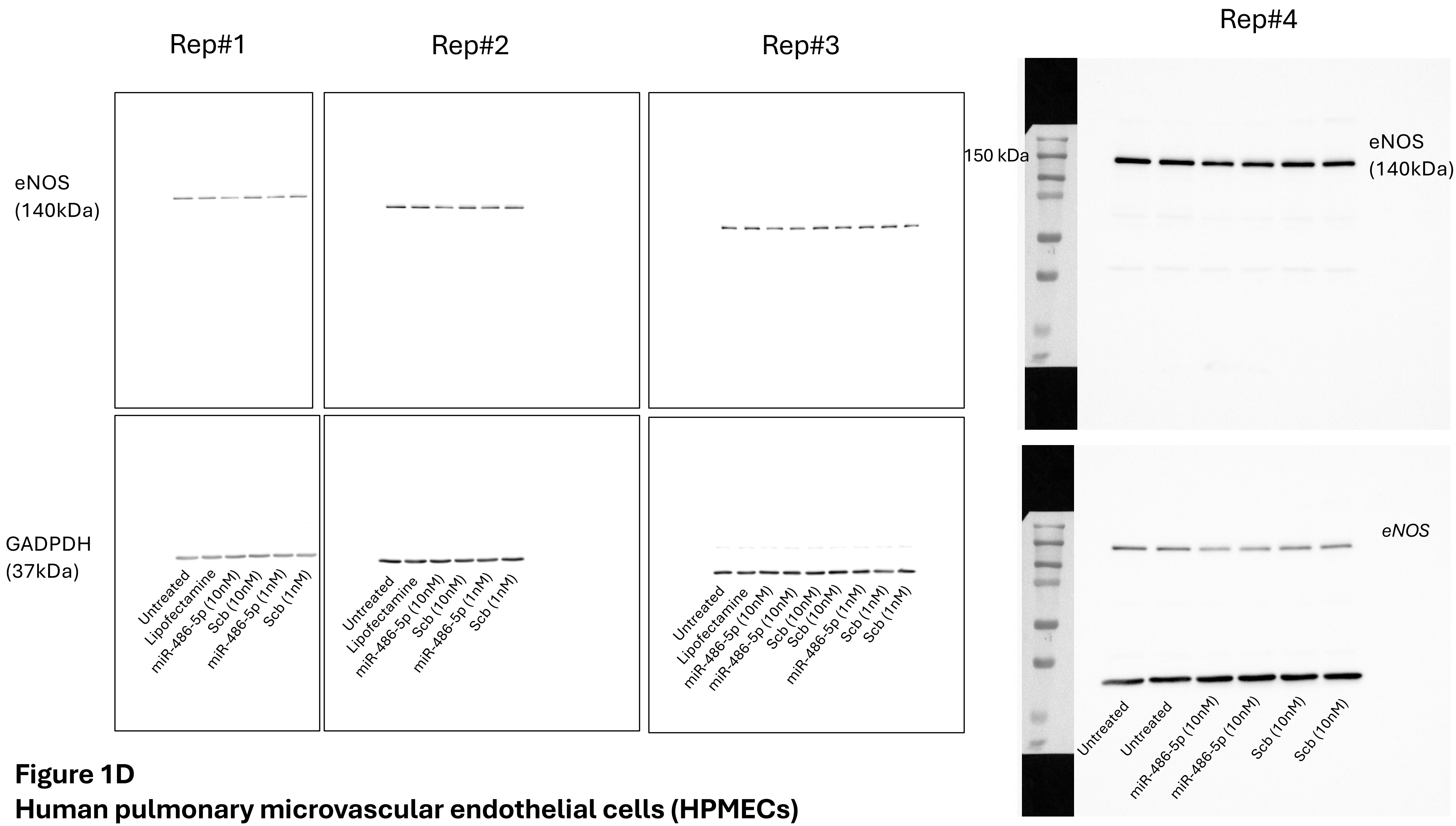

Figure 1D
Human pulmonary microvascular endothelial cells (HPMECs)

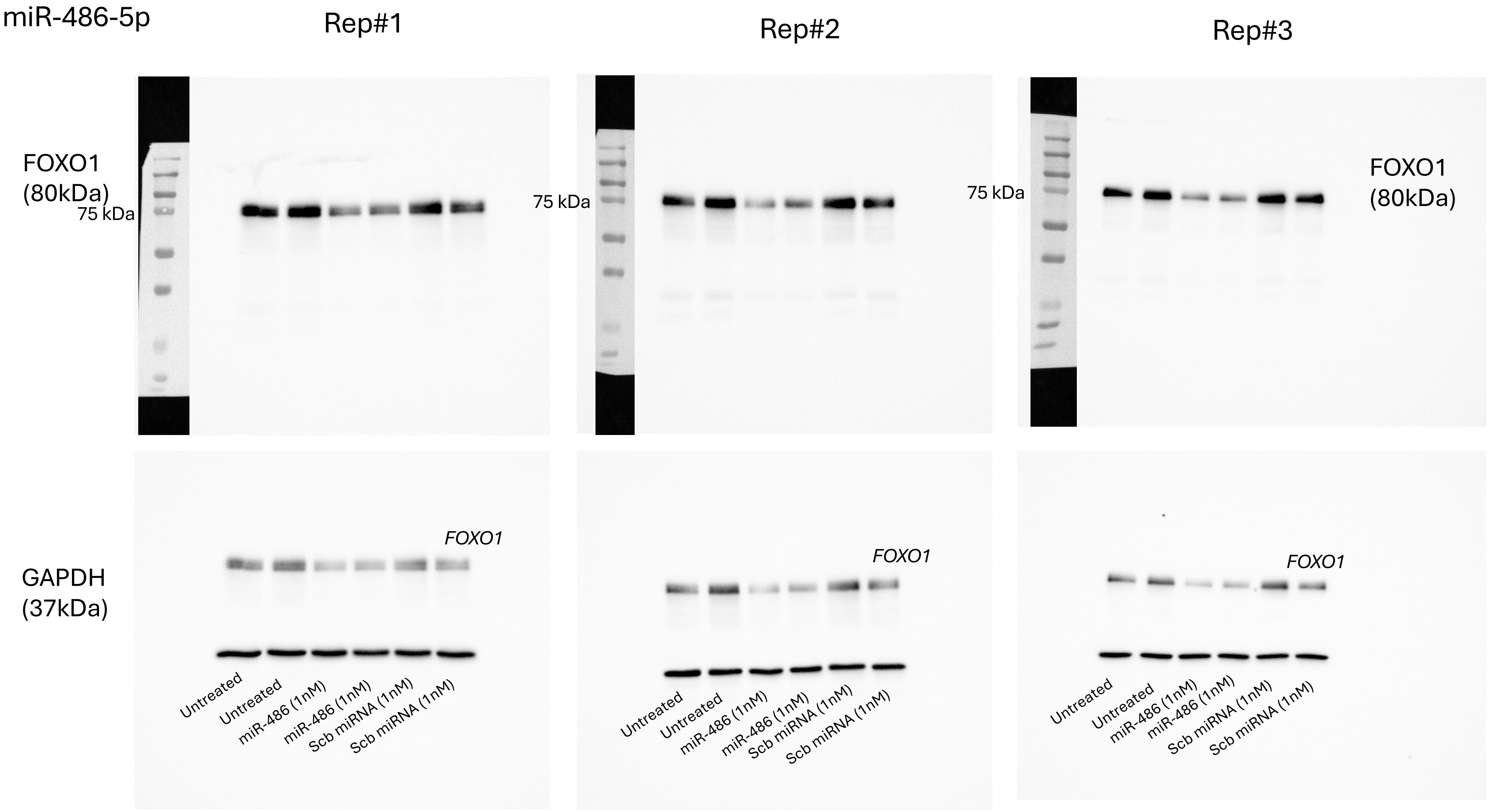
miR-486-5p
Rep#1
Rep#2
Rep#3
FOXO1 (80kDa)
75 kDa
75 kDa
75 kDa
FOXO1 (80kDa)
GAPDH (37kDa)
FOXO1
FOXO1
FOXO1
Untreated
Untreated
miR-486 (1nM)
miR-486 (1nM)
Scb miRNA (1nM)
Scb miRNA (1nM)

Figure 7A

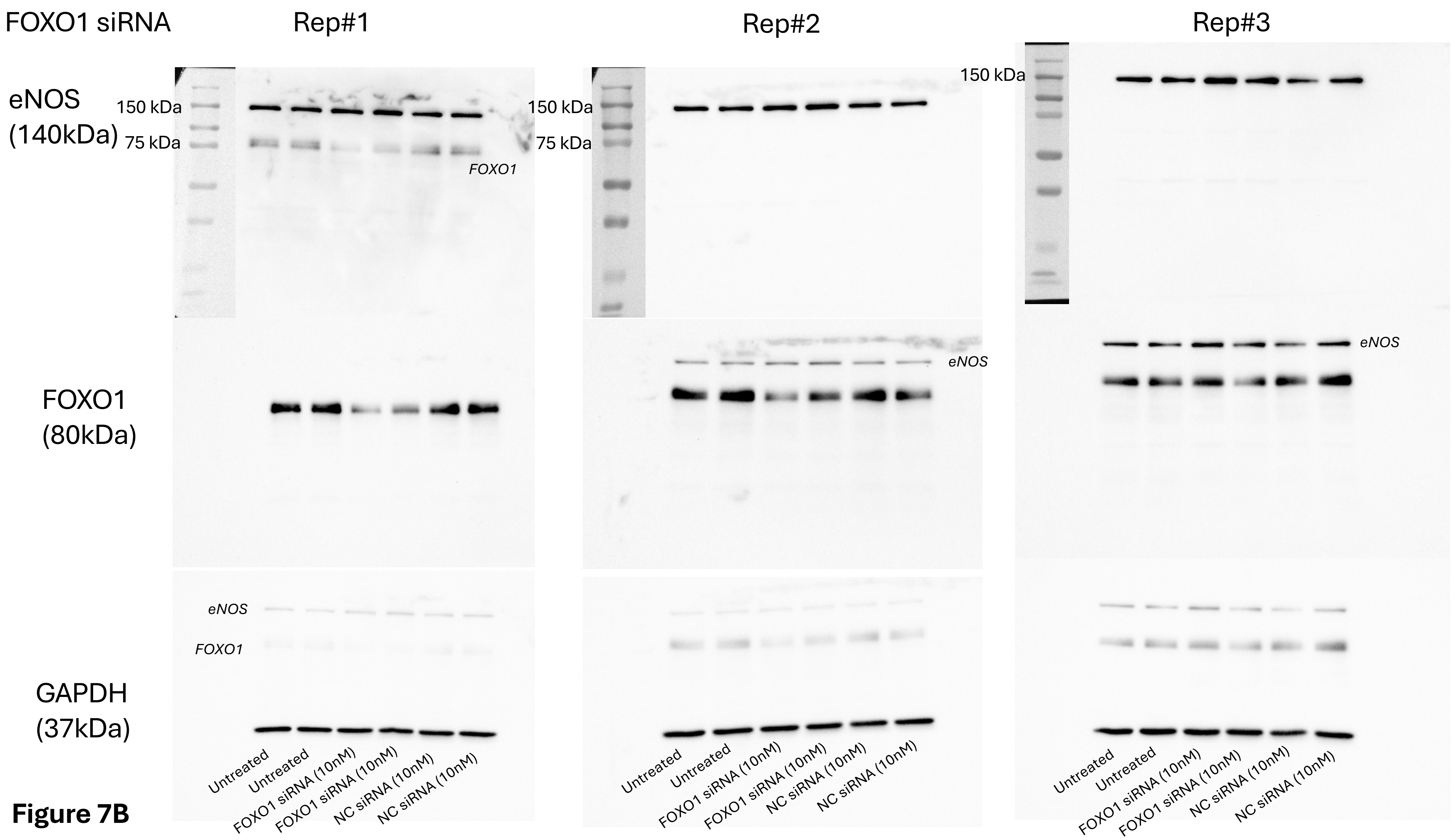
FOXO1 siRNA
Rep#1
Rep#2
Rep#3
eNOS
(140kDa)
FOXO1
(80kDa)
GAPDH
(37kDa)
150 kDa
75 kDa
FOXO1
150 kDa
75 kDa
150 kDa
eNOS
eNOS
eNOS
FOXO1
Untreated
Untreated
FOXO1 siRNA (10nM)
FOXO1 siRNA (10nM)
NC siRNA (10nM)
NC siRNA (10nM)

Figure 7B

Rep#4 blots #1, #2

eNOS
(140kDa)

FOXO1
(80kDa)

GAPDH
(37kDa)

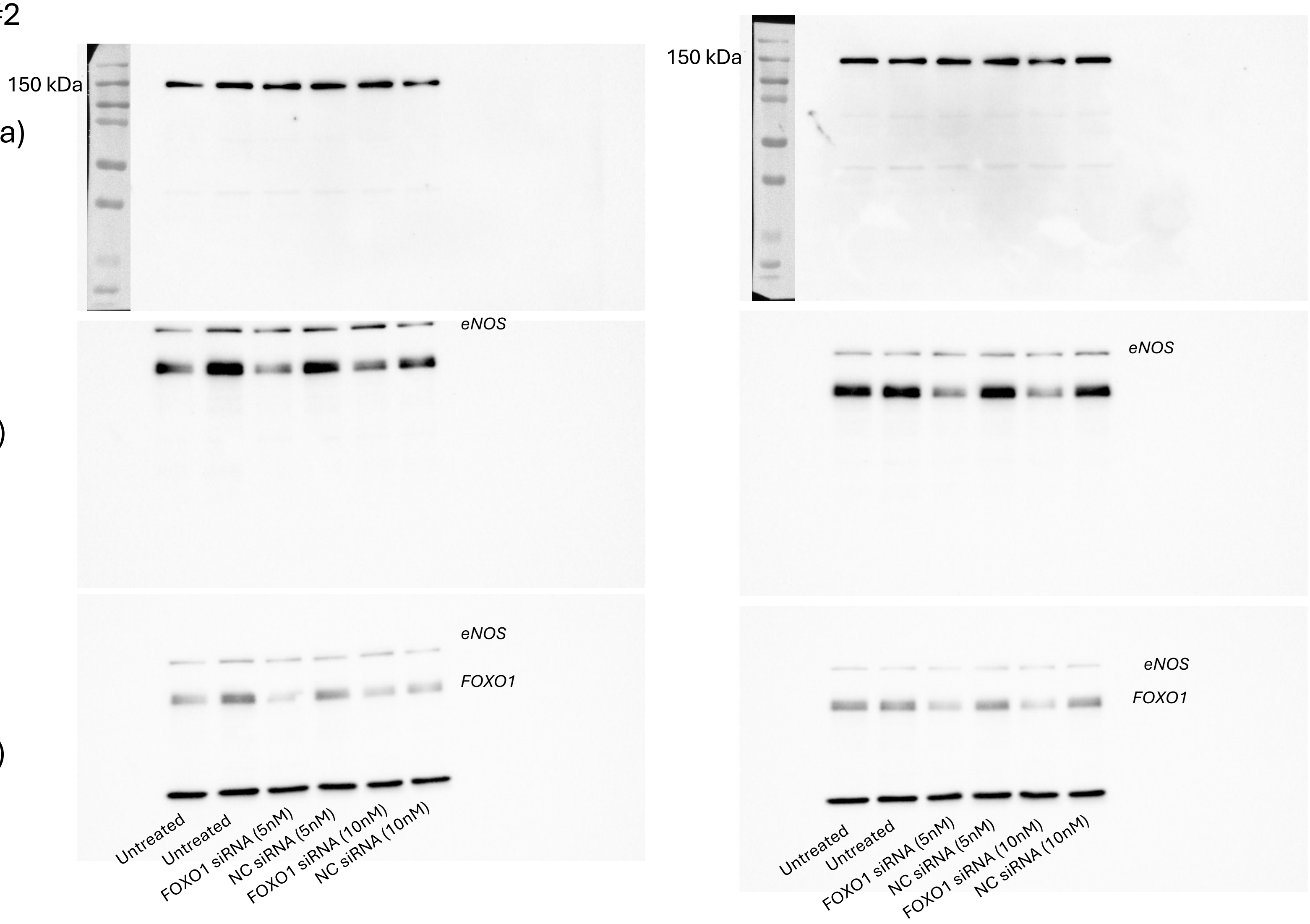

FOXO1 siRNA
Figure 7B

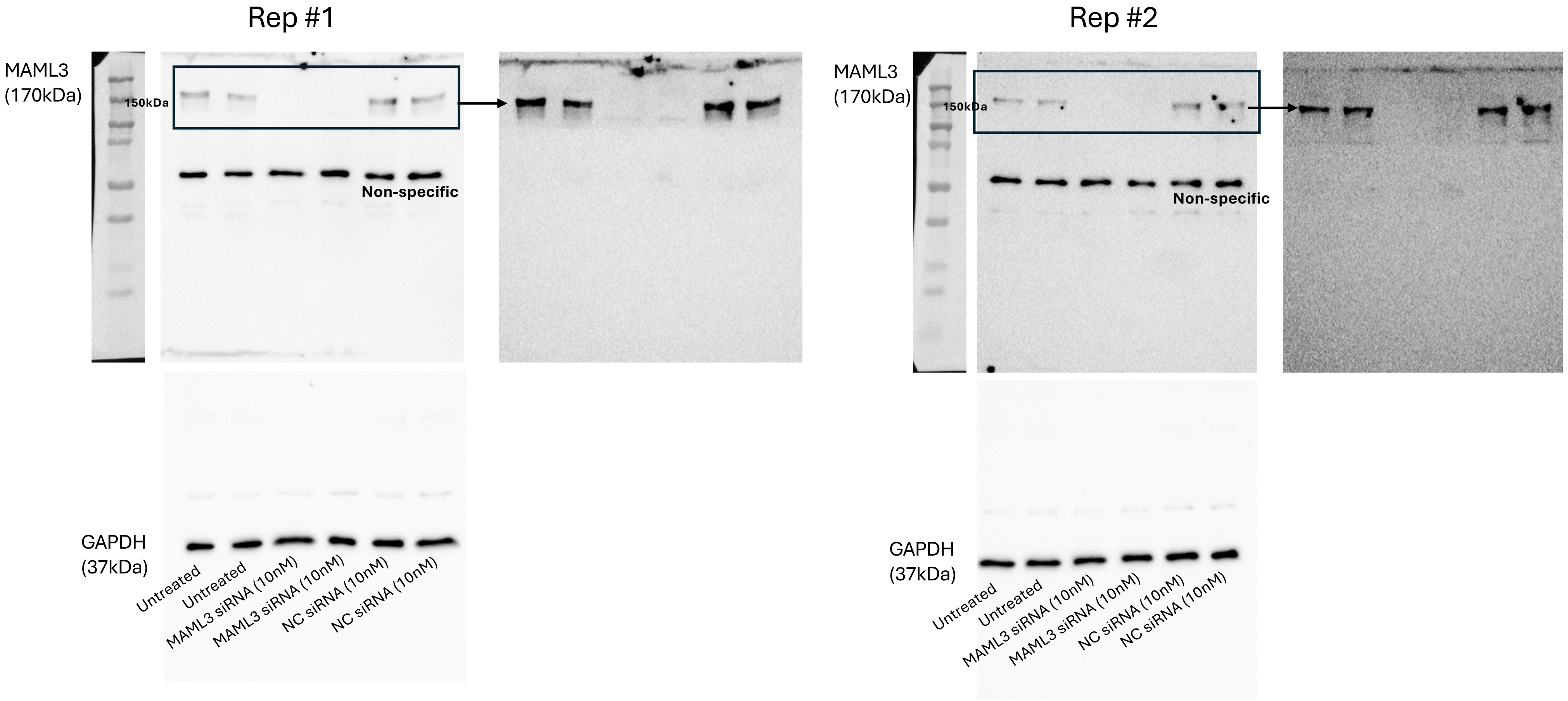

MAML3 siRNA

Figure 7D

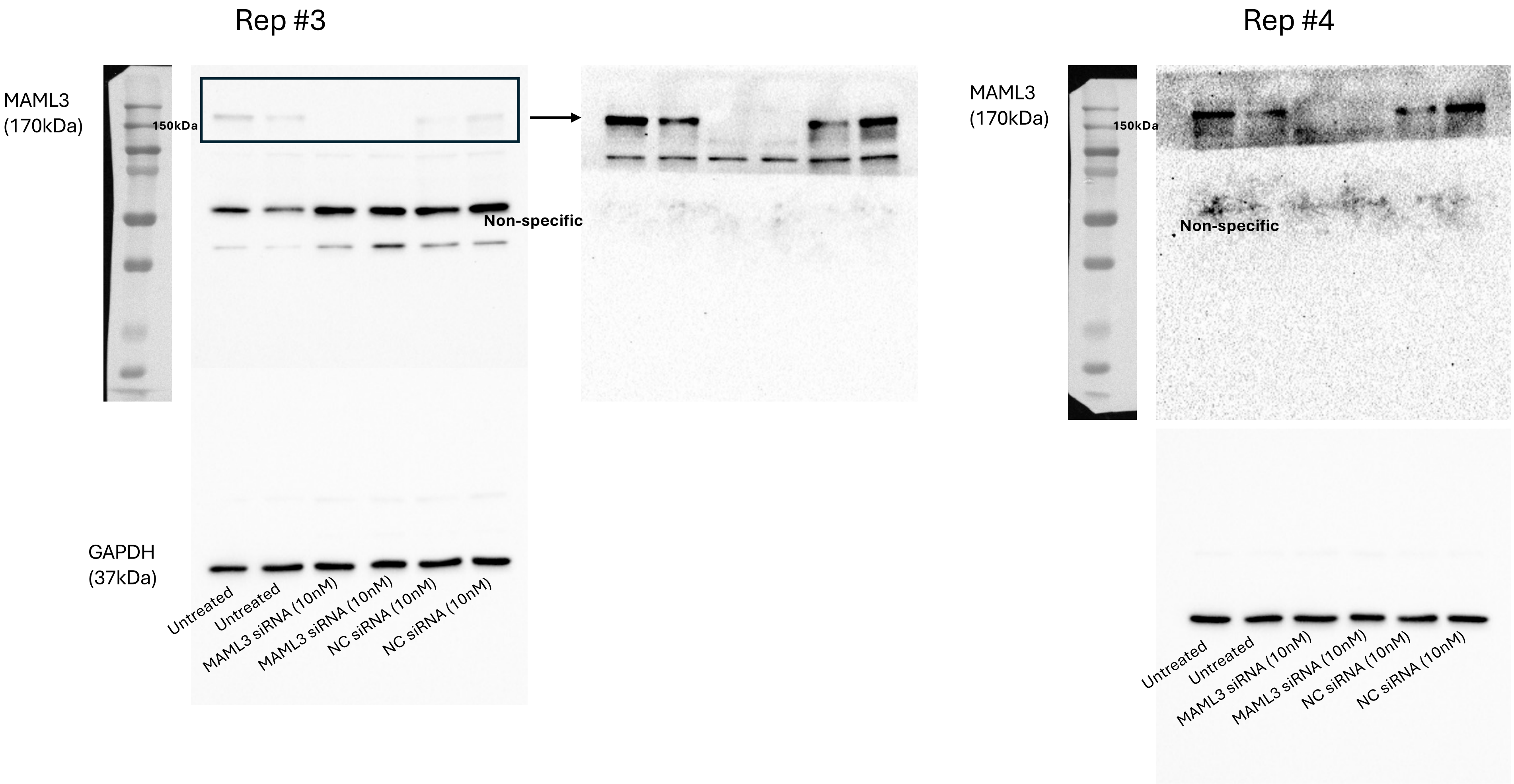

MAML3 siRNA

Figure 7D

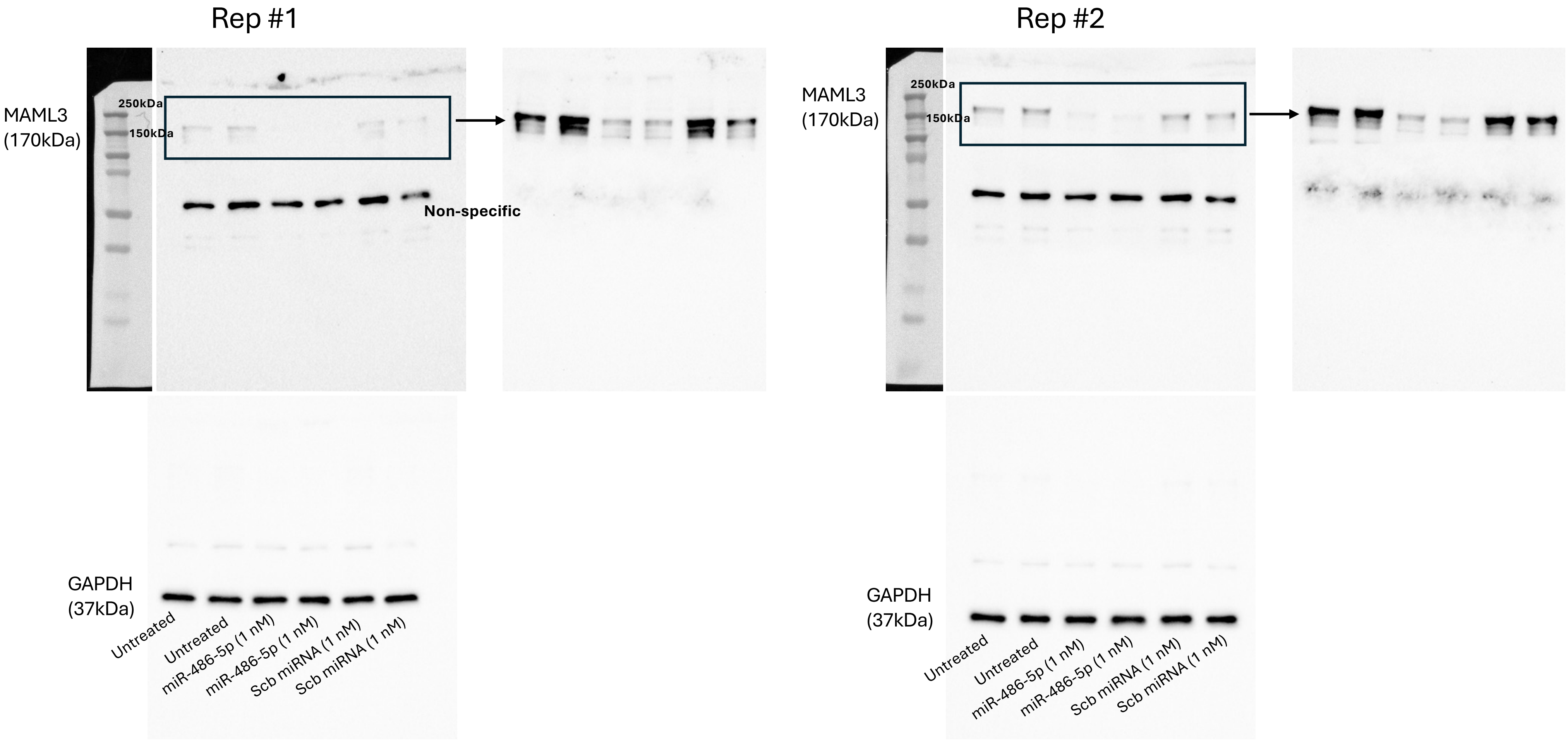

miR-486-5p

Figure 7C

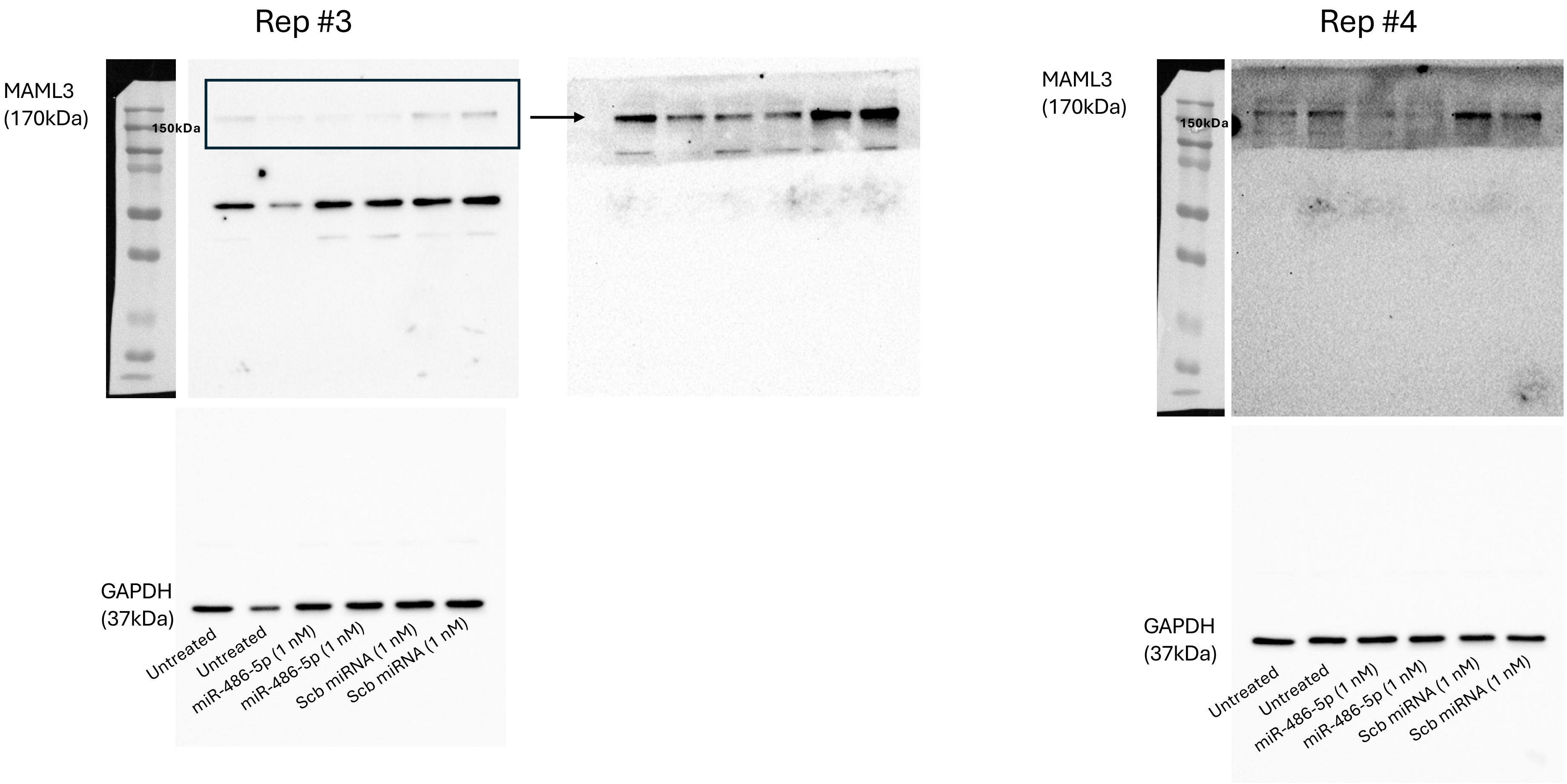

miR-486-5p

Figure 7C

Rep#1 Rep#2

eNOS
(140 kDa)

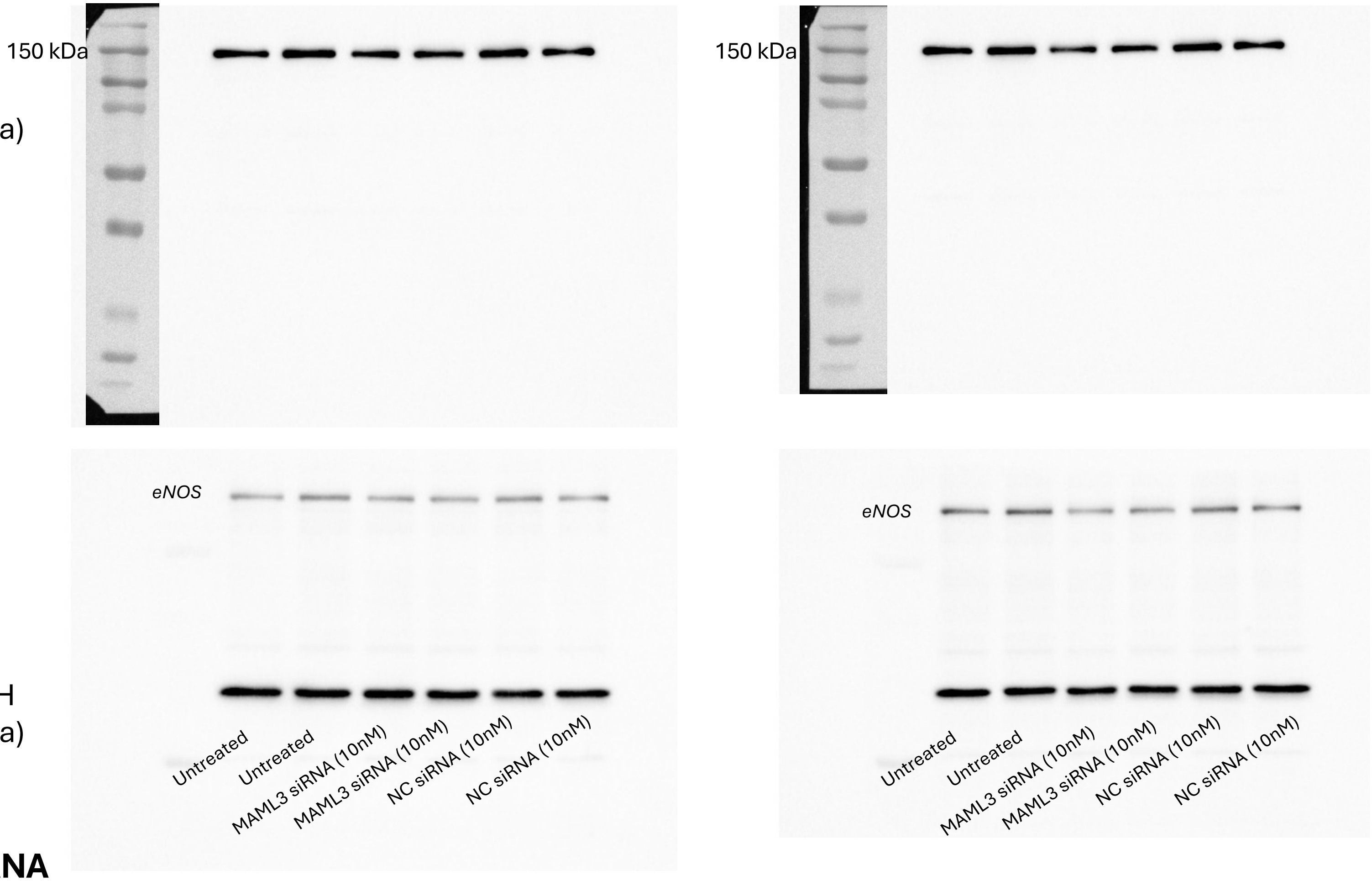

GAPDH
(37 kDa)

MAML3 siRNA
Figure 7D

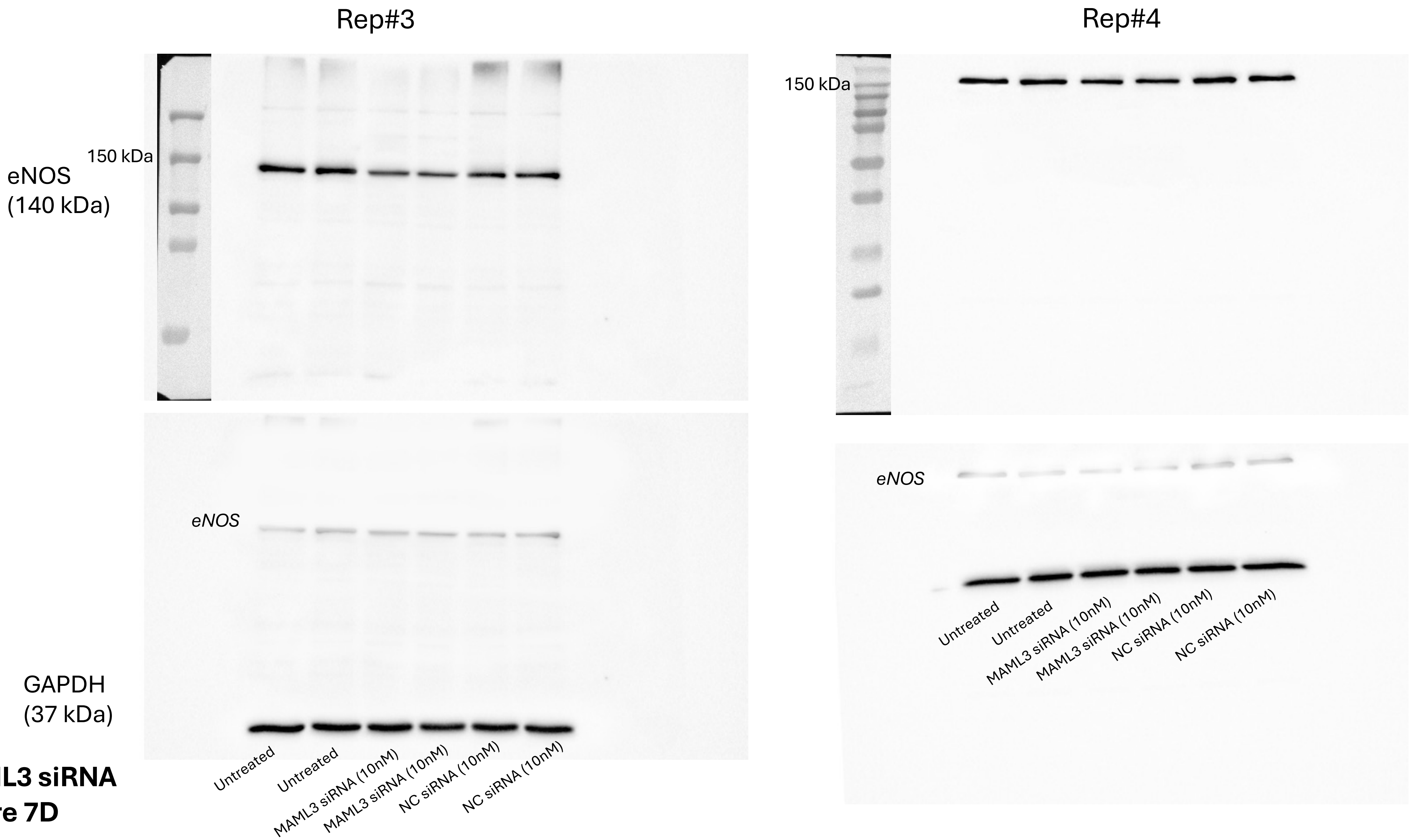

MAML3 siRNA
Figure 7D

Rep#5

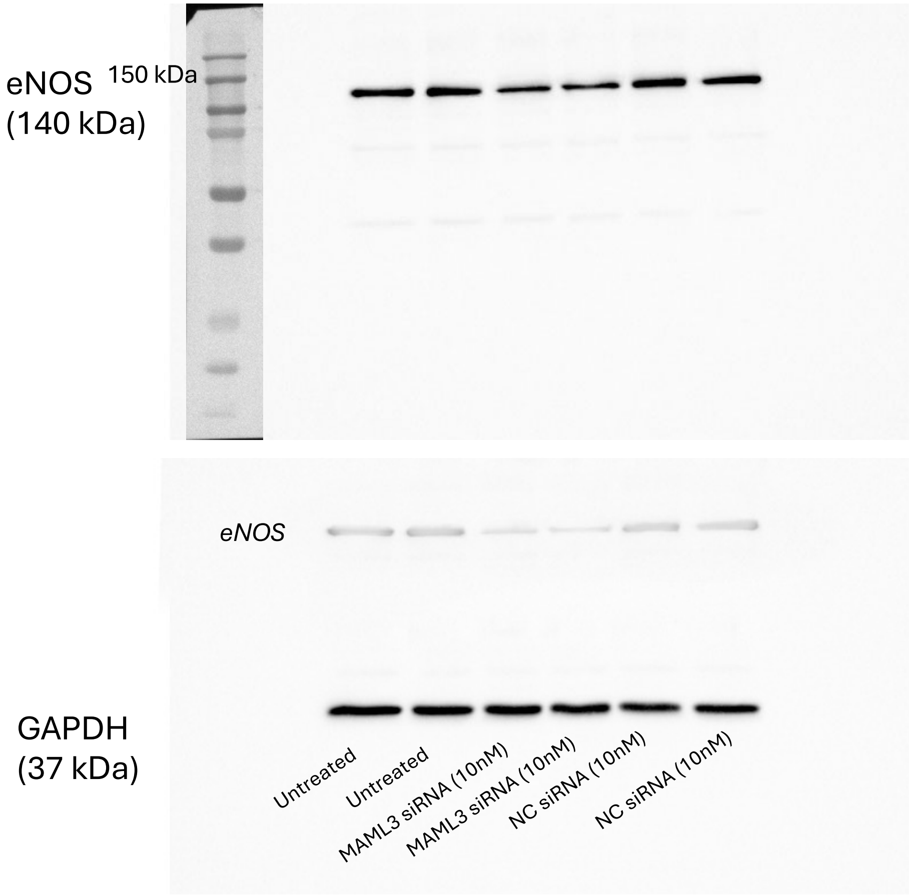

Rep#6

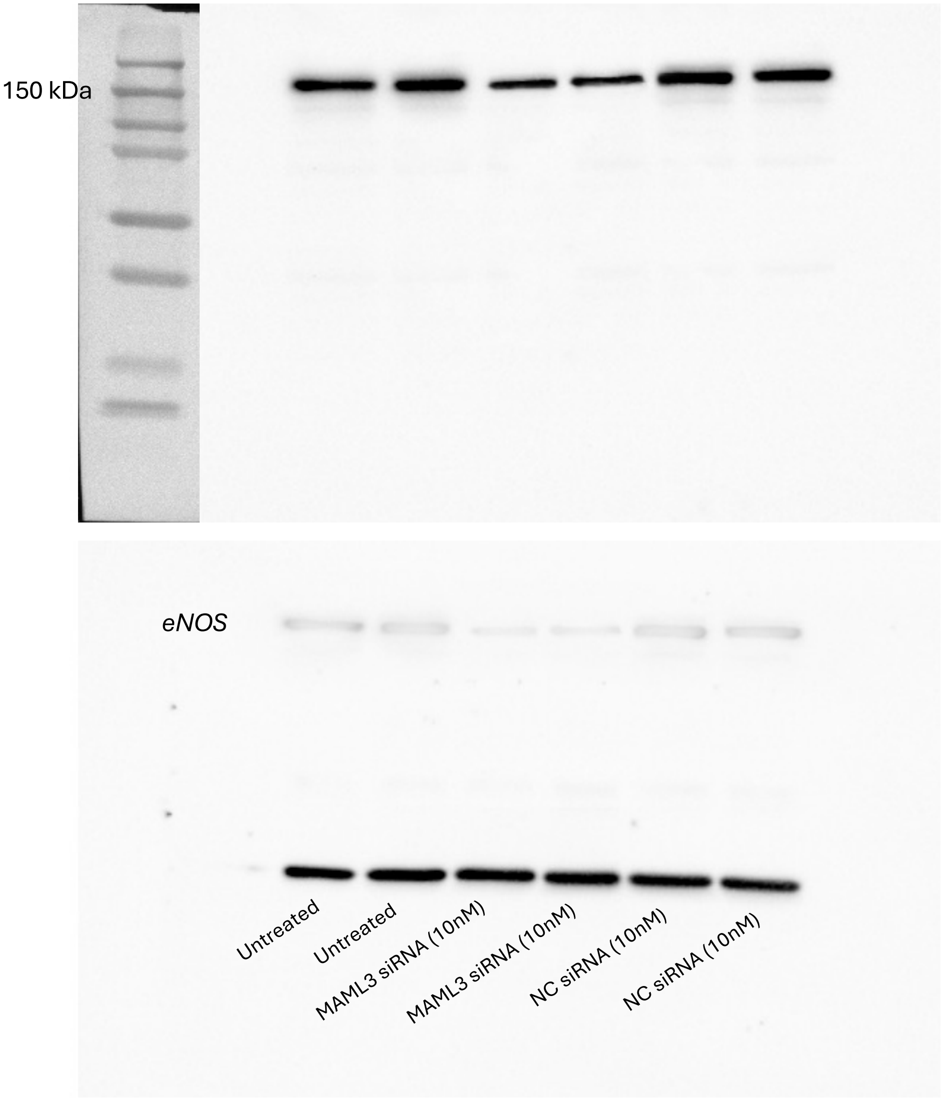

MAML3 siRNA
Figure 7D

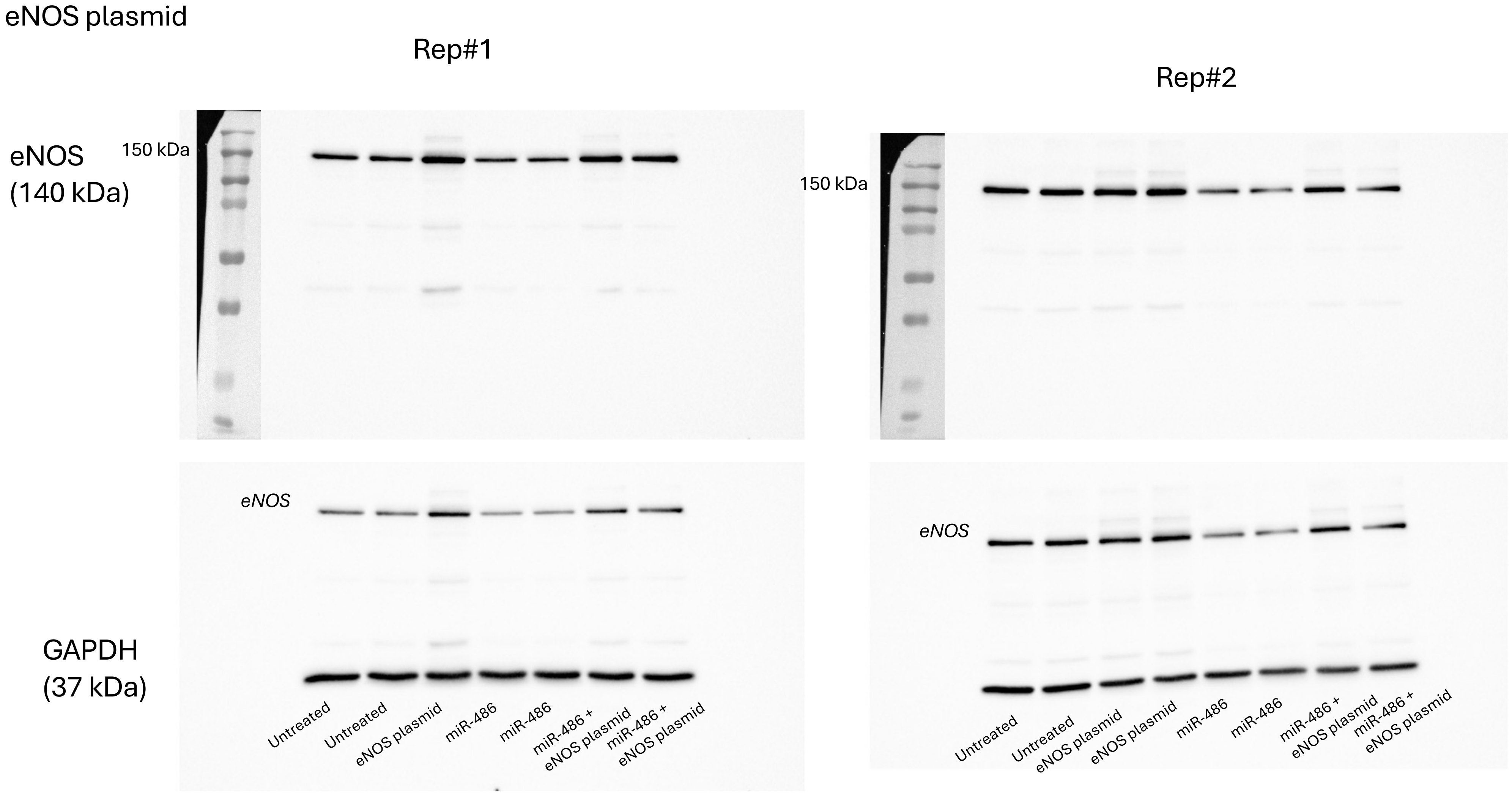

Supplemental figure 2

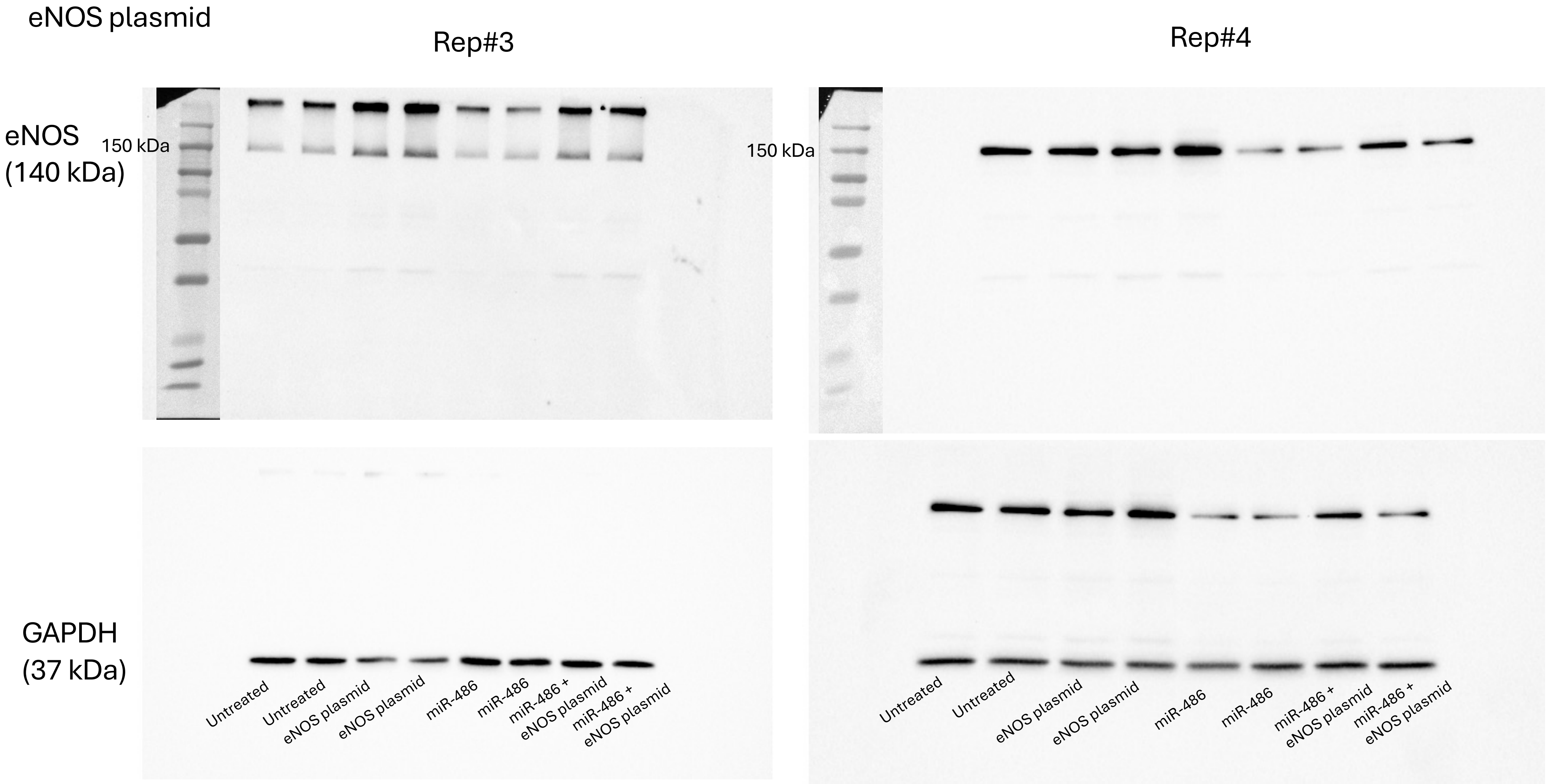

Supplemental figure 2
